# Individual transcriptomic response to strength training for patients with myotonic dystrophy type 1

**DOI:** 10.1172/jci.insight.163856

**Published:** 2023-07-24

**Authors:** Emily E. Davey, Cécilia Légaré, Lori Planco, Sharon Shaughnessy, Claudia D. Lennon, Marie-Pier Roussel, Hannah K. Shorrock, Man Hung, John Douglas Cleary, Elise Duchesne, J. Andrew Berglund

**Affiliations:** 1 RNA Institute, College of Arts and Sciences, University at Albany-SUNY, Albany, New York, USA.; 2 Department of Health Sciences, Université du Québec à Chicoutimi, Saguenay, Québec, Canada.; 3 Groupe de Recherche Interdisciplinaire sur les Maladies Neuromusculaires (GRIMN), Centre intégré universitaire de santé et de services sociaux du Saguenay-Lac-Saint-Jean, Saguenay, Quebec, Canada.; 4 Centre de recherche Charles-Le Moyne Saguenay–Lac-Saint-Jean sur les innovations en santé (CR-CSIS), Faculté de médecine et des sciences de la santé de l’Université de Sherbrooke, Site Saguenay, Saguenay, Quebec, Canada.; 5 Department of Basic Sciences, Université du Québec à Chicoutimi, Saguenay, Québec, Canada.; 6 Department of Orthopaedic Surgery Operations, School of Medicine, University of Utah, Salt Lake City, Utah, USA.; 7 College of Dental Medicine, Roseman University of Health Sciences, South Jordan, Utah, USA.; 8 Department of Biological Sciences, College of Arts and Sciences, University at Albany-SUNY, Albany, New York, USA.

**Keywords:** Muscle Biology, Therapeutics, Bioinformatics, Genetic diseases, Neuromuscular disease

## Abstract

Myotonic dystrophy type 1 (DM1), the most common form of adult-onset muscular dystrophy, is caused by a CTG expansion resulting in significant transcriptomic dysregulation that leads to muscle weakness and wasting. While strength training is clinically beneficial in DM1, molecular effects had not been studied. To determine whether training rescued transcriptomic defects, RNA-Seq was performed on *vastus lateralis* samples from 9 male patients with DM1 before and after a 12-week strength-training program and 6 male controls who did not undergo training. Differential gene expression and alternative splicing analysis were correlated with the one-repetition maximum strength evaluation method (leg extension, leg press, hip abduction, and squat). While training program–induced improvements in splicing were similar among most individuals, rescued splicing events varied considerably between individuals. Gene expression improvements were highly varied between individuals, and the percentage of differentially expressed genes rescued after training were strongly correlated with strength improvements. Evaluating transcriptome changes individually revealed responses to the training not evident from grouped analysis, likely due to disease heterogeneity and individual exercise response differences. Our analyses indicate that transcriptomic changes are associated with clinical outcomes in patients with DM1 undergoing training and that these changes are often specific to the individual and should be analyzed accordingly.

## Introduction

Myotonic dystrophy type 1 (DM1) is caused by a CTG-repeat expansion in the 3′-untranslated region of the dystrophia myotonica protein kinase (*DMPK*) gene (1–3). DM1 is the most common form of adult-onset muscular dystrophy, and the worldwide prevalence of DM1 is believed to be 1 in 8,000. However, a recent study using newborn blood spots in New York state revealed that the prevalence of DM1 was 1 in 2,100 (4), suggesting that its prevalence may be much higher than previously reported. Prevalence can also be much higher in certain geographic regions, such as Saguenay–Lac-Saint-Jean region of Québec (Canada), where it reaches 1 of 475 individuals (5). DM1 is a multisystemic disorder characterized by a wide range of symptoms such as muscle weakness and wasting, heart issues, cognitive impairment, gastrointestinal issues, and cataracts, among many health challenges (6). Symptoms are highly variable from one individual to the next, making DM1 an extremely heterogeneous disease.

In DM1, the toxic CUG-expansion RNA sequesters the muscleblind-like (MBNL) family of RNA-binding proteins, leading to a host of downstream molecular problems including defects in alternative polyadenylation, RNA localization and alternative splicing (7–10). The latter is one of the most well-studied and defining mechanisms of the disease, with misregulation of specific alternative splicing events directly linked to numerous symptoms of DM1. For example, increased inclusion of *CLCN1* exon 7A has been linked to myotonia, one of the namesake symptoms of the disease (11, 12). Other mechanisms commonly studied in the context of DM1 are increased levels of CUGBP1 (13), repeat-associated non-AUG translation (14), microRNA dysregulation (15), and the sequestration of transcription factors by the toxic RNAs (16). A lesser-studied aspect of DM1 is differential gene expression (DGE); however, dysregulation of gene expression has been identified across multiple tissues in patients with DM1 (17–20).

Due to the characteristic muscle wasting and weakness of DM1, strength training to counteract these deficiencies is a promising therapeutic strategy. Moderate-intensity strength training and aerobic training have previously been shown to be safe for patients with DM1 (21). In a study by Roussel et al., strength training was found to alleviate skeletal muscle impairments and induce lasting functional gains in men with DM1 (22). In this study, participants underwent a 12-week strength-training program, and *vastus lateralis* muscle biopsies were taken both prior to and after the training program. Given that clinical improvement was observed in these patients with DM1 after strength training, we evaluated the transcriptomic changes present in muscle samples from these patients before and after training.

## Results

### Posttraining changes in clinical measurements.

Patients previously underwent a 12-week strength-training program with muscle biopsies before and after training (22). Briefly, 11 male patients with DM1 from the Saguenay–Lac-Saint-Jean region of Québec participated in a 12-week training program consisting of a total 21 supervised training sessions. Baseline characteristics were collected for these individuals, plus 6 unaffected individuals who did not undergo training (Table 1). Following a brief low-moderate intensity aerobic warm-up, patients completed 3 series of 6–8 repetition maximum (RM) of 5 lower-limb training exercises (leg extension, leg press, hip abduction, squat, and plantar flexion) with a 3-minute rest between the series. Clinical and anthropometric measurements were performed before and after the training program by trained physiotherapists. As originally reported (22), significant improvement in strength and physical capacity was found across individuals following this program (Table 2), with the percentage of change before training to after training varying between participants. The greater percentages of improvement were found for the 1-RM measurements, with a mean ranging from 10% to 154%. The mean percentage of change for knee extensors strength, walking speeds, and 30-second sit-to-stand (30ssts) test are lower (range 1%–33%), but all participants show at least 1 clinical measure outside the standard error of measurement and exceeding the minimal detectable change (MDC) value. As previously described, these data support an overall improvement at the clinical level for all participants with DM1 following the strength-training program.

### Identification of DMPK gene expression changes following strength training.

Since strength training was clinically beneficial for the participants with DM1, we examined the effects of strength training at the transcriptomic level. To identify transcriptome changes, we performed RNA-Seq on RNA extracted from *vastus lateralis* biopsies from the participants with DM1 before and after the training program and from controls who did not undergo the training program. Samples from 2 participants (nos. 2182 and 1955) were insufficient in quality and/or quantity to generate RNA-Seq libraries and were not included. The training program was designed to target the quadriceps (22), as this large muscle group has a greater potential impact on a patient’s daily function by exerting actions on both knee and hip joints (23, 24). As such, the *vastus lateralis*, the largest of the 4 muscles in the quadriceps femoris muscle, was selected for biopsy and transcriptomic analysis.

**Figure 1:**
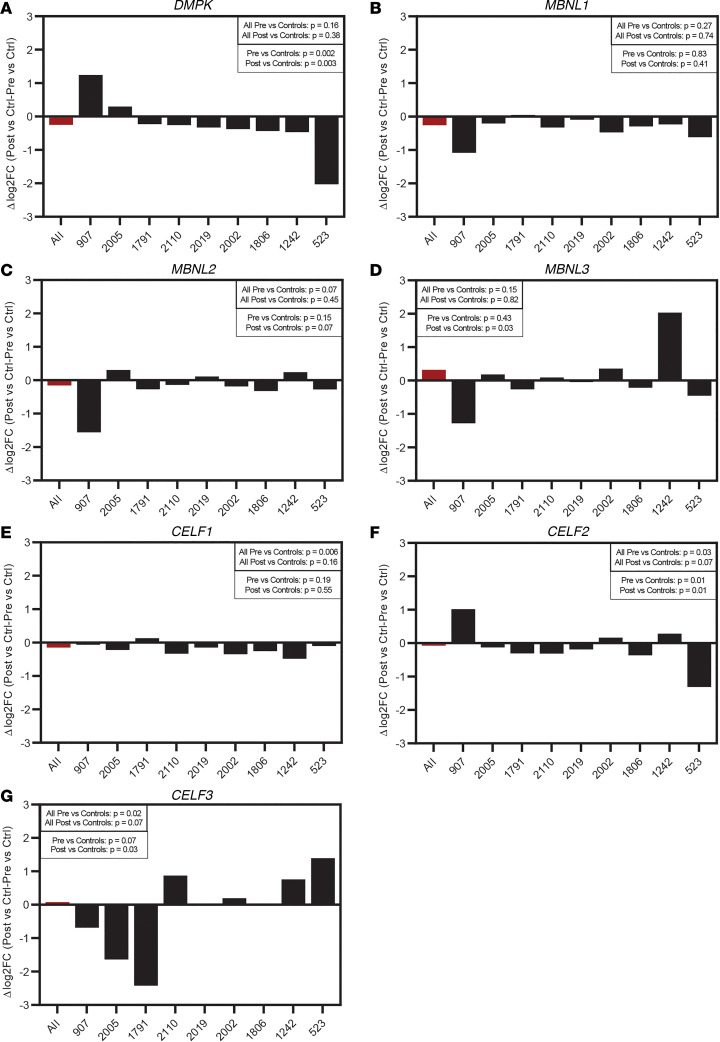
Expression changes in DM-related genes after strength training. (**A**–**G**) The change in expression, measured as log_2_FC from pretraining to posttraining in individuals as compared with unaffected controls from RNA-Seq data, for each study participant for the following DM1-related genes: (**A**) *DMPK*; (**B**) *MBNL1*; (**C**) *MBNL2*; (**D**) *MBNL3*; (**E**) *CELF1*; (**F**) *CELF2*; and (**G**) *CELF3*. Values are shown for both individual analysis (black bars) and grouped analysis (red bar). *P* values were calculated using a quasi-likelihood F test to test for differential expression among pretraining individuals, posttraining individuals, grouped pretraining individuals, and grouped posttraining individuals.

The participants for this training program showed considerable clinical heterogeneity in terms of CTG repeat length (67–1,200 repeats), BMI (22.6 to 38.6 kg/m^2^), and age (31 to 60 years), among other clinical differences (Table 1). Due to disease heterogeneity and individual differences in response to exercise, we examined expression changes in genes important in DM1 (*DMPK, MBNL1-3,* and *CELF1-3*) both grouped and individually (Figure 1). Grouped analysis provided quantification of average expression in the samples. For grouped analysis, all pretraining samples were included in one experimental group, and all posttraining samples were included in another experimental group. The expression changes in each of the experimental groups were compared with the controls (i.e., the unaffected samples held at baseline) using quasi-likelihood F test. The difference in the log_2_ fold change (FC) for posttraining samples compared with controls and for pretraining samples compared with controls was then calculated and reported as Δlog_2_FC. The same approach was taken for analyzing each individual separately. Interestingly, we observed substantial changes in *DMPK* expression after the strength-training program when samples were examined individually but not when samples were grouped (Figure 1A). While most individuals demonstrated a decrease in *DMPK* expression, 2 individuals (907 and 2005) experienced an increase in *DMPK* expression (Figure 1A). We also looked at changes in the expression of *MBNL1/2/3* and *CELF1/2/3* previously implicated in DM1 disease mechanism (Figure 1, B–G). As with *DMPK*, changes in expression of these transcripts were minimal when examining the average effect from the grouped analysis. The observed changes in *MBNL* mRNA levels do not necessarily represent comparable changes in protein levels because changes in protein expression do not always correlate with changes in mRNA levels; it is also possible that the additional MBNL proteins could become sequestered through interactions with expanded *DMPK* RNA.

Changes across these transcripts were modest for most individuals, apart from individuals 907 and 523, who also showed the largest changes in *DMPK* expression (Figure 1A). These data suggest, at the individual level, that transcriptomic changes are associated with strength training. Our study is the first to our knowledge to demonstrate that strength training has an effect of reducing *DMPK* transcript levels in patients with DM1 on an individual level. Interestingly, a recent study by Mikhail et al., reported no changes in *DMPK*, *MBNL1*, or *CELF1* transcript levels after 12 weeks of moderate-intensity cycling training, and only MBNL2 protein levels increased (25). While individual-level analysis of their data (Supplemental Figure 1; supplemental material available online with this article; https://doi.org/10.1172/jci.insight.163856DS1) does show *DMPK* differences for specific individuals, the response is very heterogenous and mostly results in slight upregulation. Differences in training type (strength vs aerobic) and sample population between the 2 studies may underlie the observed difference in gene expression following training. Furthermore, to better understand potential population differences between these studies, we compared 3 DM1-associated splicing events (*ATP2A1* exon 22, *CLASP1* exon 20, and *CLCN1* exon 7a) for control participants, pretraining participants, and posttraining participants with DM1 with published data on control and pretraining and posttraining individuals with DM1 (17). Analyses across these 3 events (Supplemental Figure 2) show that the participants in the aerobic training group had greater missplicing at the start of the study.

### Strength training in individuals with DM1 affects expression of genes previously implicated in transcriptomic response to exercise.

While evaluating changes at an individual level revealed genes that may be uniquely rescued in a single individual, we also looked at whether genes previously shown to change in response to exercise were also changed after strength training. From our data, we identified genes published in Amar et al. (26) that change in response to long-term resistance exercise training. This study was a meta-analysis of 43 human exercise training studies, with 3 that shared our strength-training program criteria of 12-week resistance exercise training with *vastus lateralis* muscle biopsies performed before and after exercise. Seven genes differentially expressed in response to long-term resistance exercise in the meta-analysis also exhibited changes in expression after strength training in our study (Supplemental Figure 3). These expression changes occurred in the same direction for most individuals in our program as in Amar et al. (26), with 6 extracellular matrix genes being upregulated and 1 gene that is reported to negatively regulate skeletal muscle hypertrophy being downregulated (Supplemental Figure 3). Together these data validate that individual analyses can recapitulate known transcriptomic responses to exercise training previously seen in global analyses of control populations.

### Strength training alters splicing in DM1.

Alternative splicing dysregulation is a hallmark molecular feature of DM, with some splicing events closely connected to clinical outcomes (10, 11). Given the heterogeneity in clinical response and change in genes involved in DM1, we examined the starting heterogeneity in skipped exon events among participants by comparing each pretraining sample with controls for overlaps in significant skipped exon events (ΔPSI > |0.2| and FDR < 0.05) (Supplemental Figure 4A). In line with our other observations, there was considerable heterogeneity in the significant skipped exon events prior to the strength-training program in each individual as compared with controls, with only 3 events shared across all 9 individuals (Supplemental Figure 4A).

Next, we determined whether strength training had an effect at the level of alternative splicing by analyzing global splicing dysregulation before and after strength training in the participants with DM1 compared with unaffected controls. To quantify improvement in splicing, we first calculated the percentage of skipped exon events that shifted toward control levels (i.e., rescued) after the training program. Percent rescue values were calculated for the significant skipped exon events (ΔPSI > |0.2| and FDR < 0.05) that were misspliced in an individual prior to the training program. These events are categorized as DM1-specific skipped exon events. Rescued events were defined as those events where the percent spliced in (PSI) value shifted more than 10% but less than 110%, which represents an improvement or rescue in missplicing toward controls. Overrescued events were defined as a percent rescue greater than or equal to 110% beyond control levels. Misrescued events were defined as percent rescue less than 10% in the direction opposite of control levels. Using these definitions, we examined the splicing dysregulation before and after strength training in the participants with DM1.

**Figure 2:**
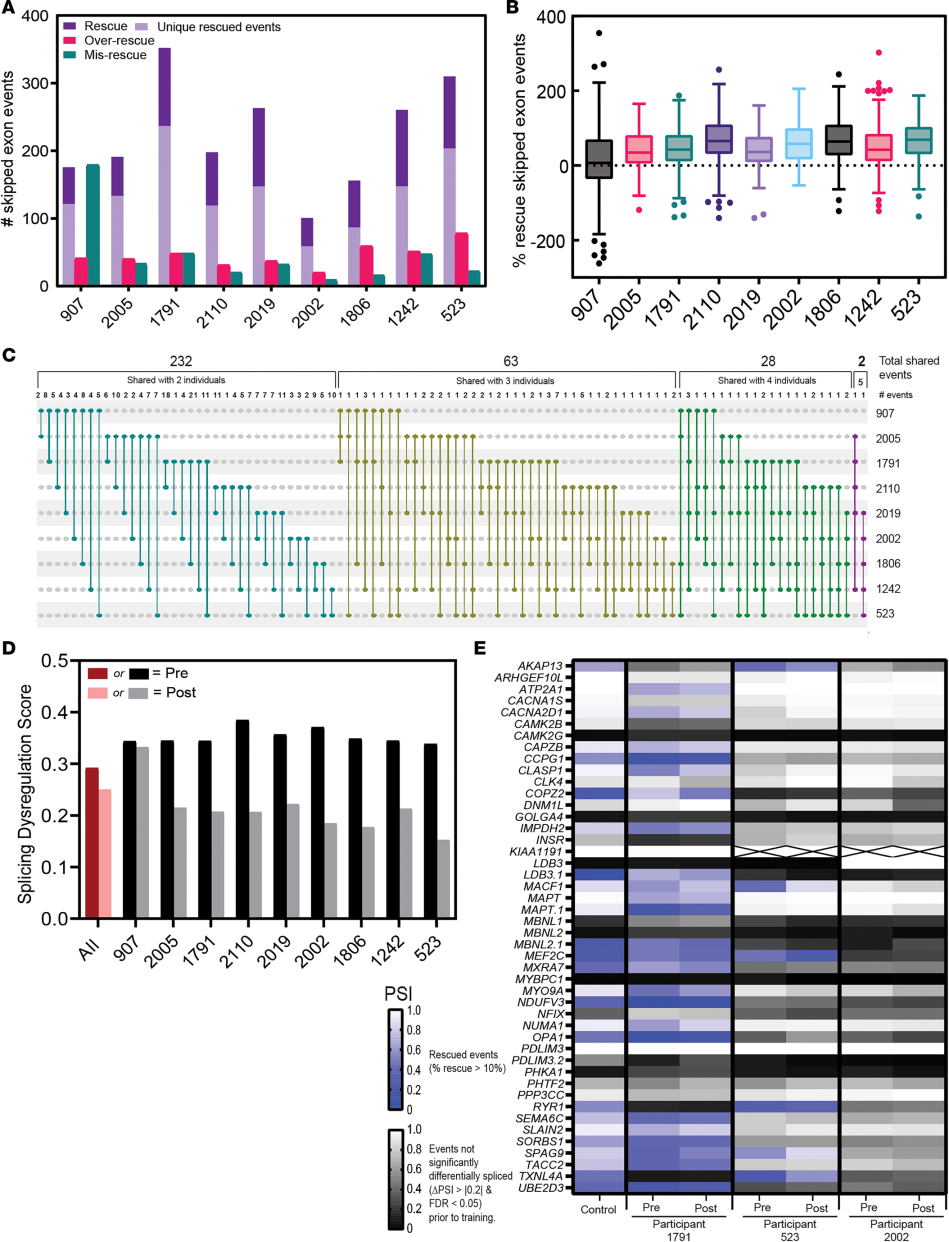
Individual improvement at the level of alternative splicing after strength training. (**A**) Number of significant skipped exon events (ΔPSI > 0.2 and FDR < 0.05) of each participant for rescued, overrescued, or misrescued events. Rescued (purple), 10% < PSI < 110%; overrescued (red), PSI ≥ 110% control; and misrescued (green), PSI < –10% opposite direction of control. Rescued events unique to each individual are indicated with light purple. (**B**) Range of percent rescue values, calculated as: (ΔPSI Pre-Post/ΔPSI Pre-Control) ***×*** 100, for all significant skipped exon events for each participant. (**C**) The overlap of shared rescued events between participants, expressed as total shared events between *n* individuals, no. of individual shared events within that total, and which individuals between whom those events are shared (connected dots). There are no shared events between 6 or more individuals. (**D**) Individual (black/gray) and grouped (red/pink) pretraining and posttraining splicing dysregulation scores. Splicing dysregulation scores are quantified as the average absolute ΔPSI of all events significantly misspliced prior to the strength-training program. (**E**) Heatmap of a panel of 46 skipped exon events that are good predictors of [MBNL]_inferred_ levels (28) for individual with the largest number of rescued events (participant 1791), and greatest (participant 523) and least (participant 2002) clinical improvement. See Supplemental Table 2 for all individual data. Statistically (blue shading) and not statistically (gray shading) significant events illustrated. X boxes in table indicate no reads present for the splicing event. Pre, pretraining sample; post, posttraining sample.

For every individual, except 907, most events (average of ~63%) misspliced prior to the strength-training program were rescued after the training program (Figure 2, A and B). Only a minority of events were misrescued or overrescued, suggesting improvement at the level of alternative splicing after strength training. While participant 907 had many rescued events, those events were counterbalanced by an almost equal number of misrescued events (Figure 2A). It is interesting that individual 907 had the largest increase in expression of *DMPK* (Figure 1A) and decrease in expression in *MBNL1-3* and *CELF2* (Figure 1, B–G). It is important to note that this individual did see an improvement in 1-RM measurements following training, suggesting a complex mechanistic response to strength training. These data support an improvement in alternative splicing dysregulation following strength training in our cohort. Due to limited muscle sample, 1 event (*CLASP1* exon 20) was validated by RT-PCR from 4 participants (Supplemental Figure 5A). Except for individual 523, who showed no difference between pre- to posttraining in the RT-PCR analysis, the RNA-Seq data were validated by the RT-PCR results.

Many rescued splicing events among the cohort were unique to each individual, ranging from 55% to 69% of all rescued events (Figure 2A). To better understand this distribution, we plotted the overlap of events between participants following training, and it showed that shared events dropped dramatically, from 232 events shared between any 2 individuals to only 2 events shared between 5 individuals (Figure 2C). Both the total shared events and number of individual events declined when comparing events shared with more individuals. The number of individual shared events for 2 individuals ranged from 1 to 21 events, 1 to 7 for 3 individuals, and 1 to 3 for 4 individuals (Figure 2C). Importantly not a single event was rescued across all individuals in this study, despite common shared splicing events present prior to training (Supplemental Figure 4A).

Finally, to quantify the global improvement in splicing, we calculated splicing dysregulation scores for each individual. This score was calculated as the average absolute ΔPSI for all skipped exon events significantly misspliced (ΔPSI > |0.2| and FDR < 0.05) prior to the strength-training program. This metric has previously been used to quantify global splicing dysregulation in DM (27). We found that all individuals showed a decrease in their splicing dysregulation score after the training program, albeit only to a minor extent for individual 907 (Figure 2D). These data further support an improvement at the level of alternative splicing following strength training. It is important to note that, as within individual transcript analysis (Figure 1), the small degree of improvement experienced by 907 cancels out the substantial improvement from other individuals when the scores are combined (Figure 2D). Next, we sought to determine whether any of the events known to be commonly misspliced in patients with DM1 were rescued after the strength-training program. We focused on a set of 46 splicing events previously described to be informative in predicting [MBNL]_inferred_ levels (28). Rescue was limited to only a handful of events for a few participants (Supplemental Figure 6), except for participant 1791, for whom more than half of these events were partially or fully rescued (Figure 2E). Only a handful of these events were rescued for the individuals with the greatest clinical improvement (greatest average percent change in 1-RM measurements), individual 523, and least clinical improvement, individual 2002. It is important to note that some of these 46 events for various participants did not exhibit significant differential splicing prior to the strength-training program; therefore, percent rescue values were not calculated.

Given that the sequestration of MBNL proteins is responsible for the alternative splicing dysregulation in DM1, we sought to determine if the splicing events rescued by the training program were MBNL dependent. MBNL proteins, which regulate exon splicing in a positional-dependent manner through binding YGCY motifs within or nearby the regulated exon (29, 30), are sequestered by CUG repeat RNAs that form a perfect binding motif. Skipped exon events aberrantly included in DM1 should exhibit an enrichment of YGCY motifs upstream or within the regulated exon, while events aberrantly excluded in DM1 should exhibit an enrichment downstream of the regulated exon. While an enrichment for YGCY motifs in misspliced skipped exon events in DM1 have previously been reported in numerous human tissues such as the tibialis muscle and frontal cortex of the brain (17, 27), to our knowledge, this process has not yet been examined in human *vastus lateralis*. Tanner et al. have, however, shown an enrichment of Mbnl-binding motifs in mouse quadriceps (31). To determine whether an enrichment of YGCY motifs is also present in the human *vastus lateralis*, we first calculated the log_2_(enrichment) of YGCY motifs in male DM1 *vastus lateralis* samples from DMSeq.org (17). We found that there was a modest enrichment of YGCY motifs for most male individuals with DM1 upstream of the target exon for significant inclusion events (ΔPSI > 0.2 and FDR < 0.05) and a slight enrichment of YGCY motifs downstream of the target exon for significant exclusion events (ΔPSI < –0.2 and FDR < 0.05; Supplemental Figure 7A).

We then determined whether this enrichment of YGCY motifs was present in our pretraining data. Similarly, we found that there was an enrichment of YGCY motifs upstream and within the target exon for significant inclusion events (ΔPSI > 0.2 and FDR < 0.05) before training in most individuals with DM1 (Supplemental Figure 7B). An enrichment downstream of the target exon for significant exclusion events (ΔPSI < -0.2 and FDR < 0.05) before training was not as evident. After training, however, we found that enrichment of YGCY motifs for significant inclusion events decreased in some individuals, suggesting that the splicing events rescued by the training program may be MBNL dependent. This is further supported by the enrichment of YGCY motifs upstream or within the target exon for inclusion events rescued by the training program in some individuals and the YGCY enrichment downstream of the target exon for rescued exclusion events (Supplemental Figure 7B).

### Global changes in gene expression are evident after strength training for participants with DM1 and correlate with clinical improvements.

**Figure 3:**
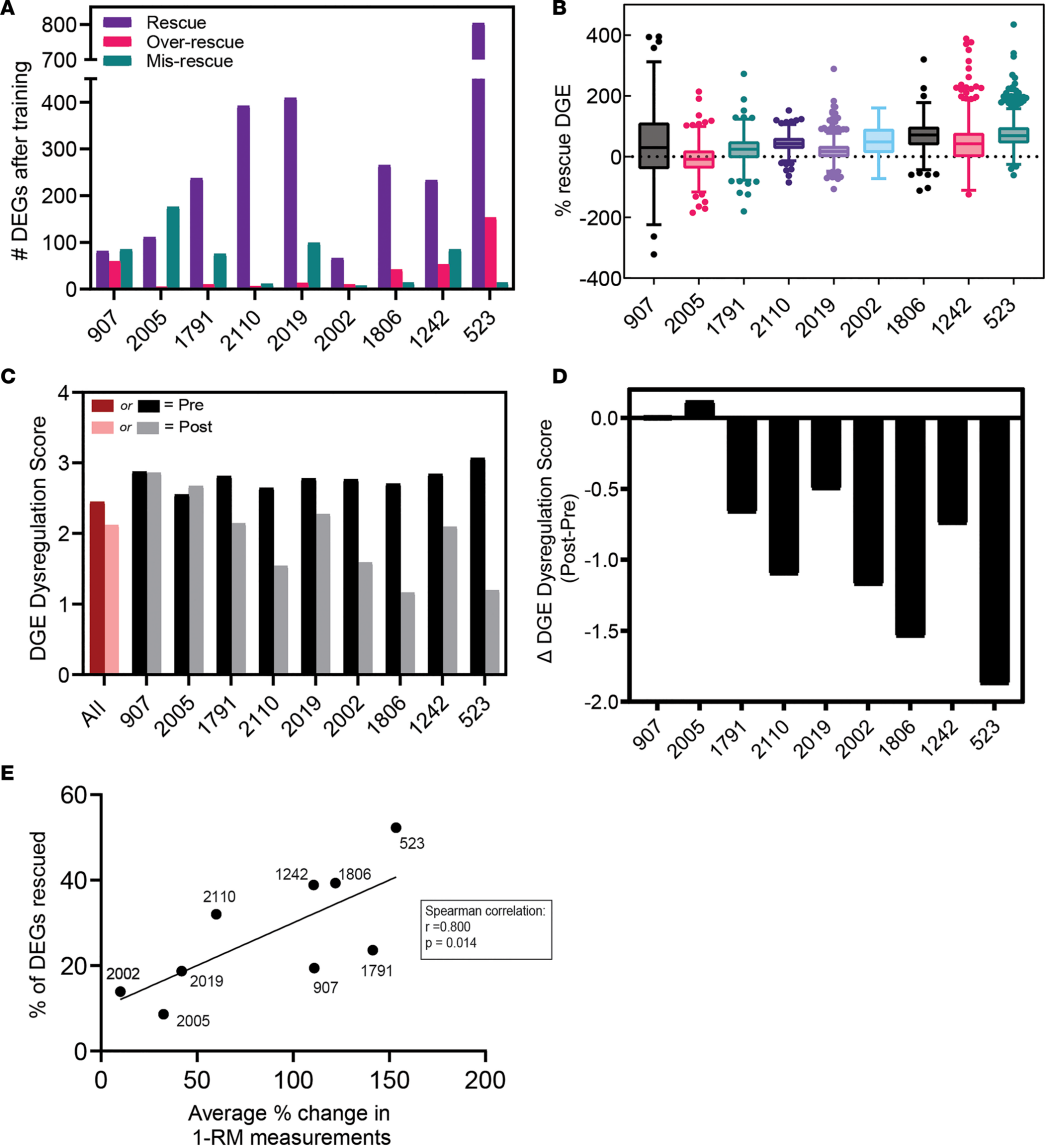
Differential gene expression changes for patients with DM1 following the strength training. (**A**) Number of DEGs rescued, overrescued, or misrescued after training. Rescued (purple), 10% < PSI < 110%; overrescued (red), PSI ≥ 110% control; misrescued (green), PSI < –10% opposite direction of control. (**B**) Range of percent rescue values, calculated as: (log_2_FC Pre-Post/log_2_FC Pre-Control) ***×*** 100, for all significant DEGs for each participant. (**C**) Individual (black/gray) and grouped (red/pink) pretraining and posttraining gene expression dysregulation scores. (**D**) Individual changes in the DGE dysregulation score after training. (**E**) Plot of percentage of rescued DEGs versus change in 1-RM clinical measurements showing Spearman correlation. DEGs, differentially expressed genes; DGE, differential gene expression; Δ, change in.

Next, we examined global changes in DGE for each individual with DM1 compared with controls by first calculating rescue percentages for each of the genes significantly differentially expressed (log_2_FC > |2| and *P* < 0.05) prior to the strength-training program (Supplemental Figure 4, B and C). We used the same cutoffs as for alternative splicing to categorize events as rescued, overrescued, or misrescued. Unlike with splicing (Figure 2A), we observed considerable variability in the improvement at the level of gene expression between individuals (Figure 3A). The range in percent rescue values (average percent rescue of gene expression) was also variable and ranged from –7% for individual 2005, to 73% for individual 523 (Figure 3B). We validated the DGE using quantitative PCR (qPCR) analysis of the *CDKN1A* gene (Supplemental Figure 5B), which showed that — except for sample 2019 — there was concordance between RNA-Seq and qPCR data. As with alternative splicing, we calculated a DGE dysregulation score to quantify global changes in gene expression for individuals with DM1 before and after the strength-training program (Figure 3C). This dysregulation score was calculated as the average absolute log_2_FC of all genes significantly differentially expressed (log_2_FC > |2| and *P* < 0.05) prior to the training program. Seven individuals showed a considerable decrease in their dysregulation score after the training program, suggesting an improvement at the level of gene expression. In contrast, 2 individuals (907 and 2005) showed minor dysregulation score changes, suggesting no amelioration of gene expression dysregulation after strength training (Figure 3D). As with alternative splicing, calculating the average dysregulation score for all participants reveals a decrease (Figure 3C) that is not reflective of the variability in gene expression changes among each individual.

Given that transcriptomic improvements in gene expression levels were evident for most individuals after strength training, we next determined if these changes correlated with clinical improvements (Table 2). To quantify and correlate clinical changes, we determined the percent change in 1-RM measurements of 4 exercises (leg extension, less press, hip abduction, and squat) after the training program and calculated the average change across these 4 measurements for each individual. To quantify changes at the transcriptome level, we calculated the percentage of differentially expressed genes (DEGs) rescued for each individual. This value was calculated as the percentage of DEGs significantly differentially expressed (log_2_FC > |2| and *P* < 0.05) before the training program, and they were then rescued (i.e., shifted toward control levels) after training. There was a robust correlation between the average percent change in 1-RM measurements and the percentage of DEGs rescued (*r* = 0.800, *P* = 0.014; Figure 3E). Given the difference in the average age between controls and participants with DM1 (32 and 48, respectively), we also recalculated the correlation while controlling for age, and this further strengthened the correlation (*r* = 0.837 *P* = 0.010; Supplemental Figure 8). Comparatively, an analysis between the level of splicing (rescued and overrescued events) and clinical improvements did not show a statistically significant correlation (Supplemental Figure 9). Taken together, these data suggest that global changes in DGE, at the individual level, correlate with clinical improvements following strength training.

### Grouped analysis masks changes in splicing and gene expression present at an individual level.

**Figure 4:**
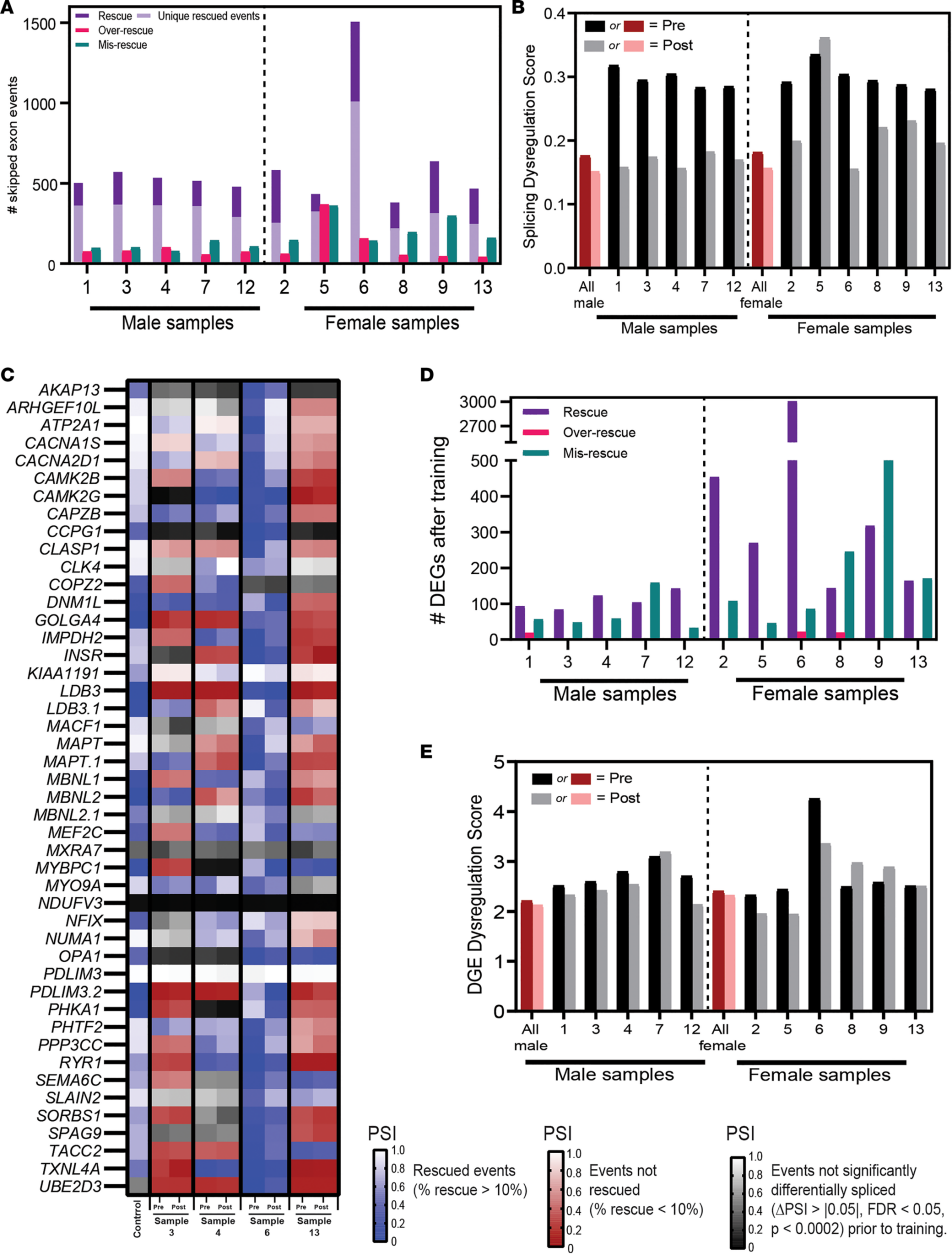
Individual transcriptomic improvements in Mikhail et al. aerobic cycling training data. Reanalysis of Mikhail et al cycling training (25) RNA-Seq data via individual analysis approach. (**A**) Number of skipped exon events rescued, overrescued, or misrescued after the cycling training program (ΔPSI > |0.05|,FDR < 0.05, *P* < 0.0002) for each participant. Rescued (purple), 10% < PSI < 110%; overrescued (red), PSI ≥ 110% control; misrescued (green), PSI < –10% opposite direction of control. Rescued events unique to individual are indicated with light purple. (**B**) Individual (black/gray) and grouped (red/pink) pre- and posttraining splicing dysregulation scores. Splicing dysregulation scores are quantified as the average absolute ΔPSI of all events significantly misspliced prior to the strength-training program. (**C**) Heatmap of a panel of 46 skipped exon events that are good predictors of [MBNL]_inferred_ levels (28) for participants with smallest (sample 6), median (sample 13), and greatest (samples 3 and 4) baseline FEV_1_ values. Statistically significant rescued (blue shading), not rescued (red shading), and not statistically significant (gray shading) events are illustrated. (**D**) Number of DEGs rescued, overrescued, or misrescued after aerobic training. Rescue was calculated as: (log_2_FC Pre-Post/log_2_FC Pre-Control) ***×*** 100. (**E**) Pre- and posttraining DGE dysregulation scores for each individual, as well as individuals grouped together. PSI, percent spliced in; DEGs:,differentially expressed genes; DGE, differential gene expression; 1-RM, 1 repetition maximum.

The recent aerobic exercise study (25) reported no splicing rescue after cycling aerobic training by participants with DM1. In that study, transcriptomic changes were measured using a grouped analysis, which our observations show can mask individual significant transcriptomic improvement (Figure 2D and Figure 3C). Reanalysis of the cycling aerobic data using an individual approach shows considerable rescue at the level of splicing following aerobic training (Figure 4A). Since our data were generated only with male participants, we reanalyzed the cycling training data (25) for male and female participants separately using the cutoffs for significance (ΔPSI > |0.05|, FDR < 0.05, *P* < 0.0002 for splicing; log_2_FC > |1.5|, *P* < 0.005 for gene expression). As with our data, the splicing dysregulation score following aerobic training was reduced for every participant after training, except participant 5 (Figure 4B). It was noted that participant 5 only participated in 50% of the cycling training sessions (25), and this could explain the lack of decrease in splicing dysregulation score compared with other participants. As with our observations, calculating the splicing dysregulation score for all male or all female participants using a grouped analysis resulted in only a subtle decrease in the splicing dysregulation score (Figure 4B). Like strength training, many rescued skipped exon events following aerobic training were unique to each individual (44%–73%; Figure 4A), further supporting heterogeneity in the response to training. We looked at the panel of 46 splicing events described in Wagner et al. (28) and found a similar heterogeneous pattern of response (Figure 4C). In general, more events were rescued across individuals than in the strength-training program, albeit with only a handful of events rescued across many individuals (Supplemental Figure 10). Again, 1 participant, sample 6, exhibited rescue of more than half of the 46 events, while most participants only saw rescue of a handful to none of these events (Supplemental Figure 10), further underlining the heterogeneity in the transcriptomic response. We examined this panel across participants who experienced a range of clinical benefit (Figure 4C), based on the forced expiratory volume in 1 second (FEV_1_), which was previously observed (25) to be a good predictor of clinical improvement after cycling training. In Mikhail et al. (25), participants with the lowest baseline FEV_1_ experienced the greatest improvement in FEV_1_ after cycling training. Using the FEV_1_ data, we plotted splicing rescue for participants with the smallest (sample 6), median (sample 13), and largest (samples 3 and 4) baseline FEV_1_ (Figure 4C). While the participant with the lowest baseline FEV_1_ (sample 6) was the individual with the largest number of panel events rescued, this trend did not hold true across the rest of the individuals.

We employed the same individual approach for analyzing gene expression level changes in the cycling training data from Mikhail et al. (25). Using this approach, we identified rescue in gene expression levels (Figure 4D) and gene expression dysregulation scores (Figure 4E) in some participants. Only slight differences in gene expression were originally reported after training (25), and these differences match the grouped gene expression dysregulation score for all participants (Figure 4E). Interestingly, when examined individually, there are a handful of participants who experienced more pronounced improvement at the level of gene expression after cycling training. For example, all but 3 participants had decreased gene expression dysregulation scores following training (Figure 4E). The 3 individuals (individuals 7, 8, and 9) with increased gene expression dysregulation scores also had a high number of misrescued events.

### The change in expression of several genes correlate with global transcriptomic changes and clinical improvements.

**Figure 5:**
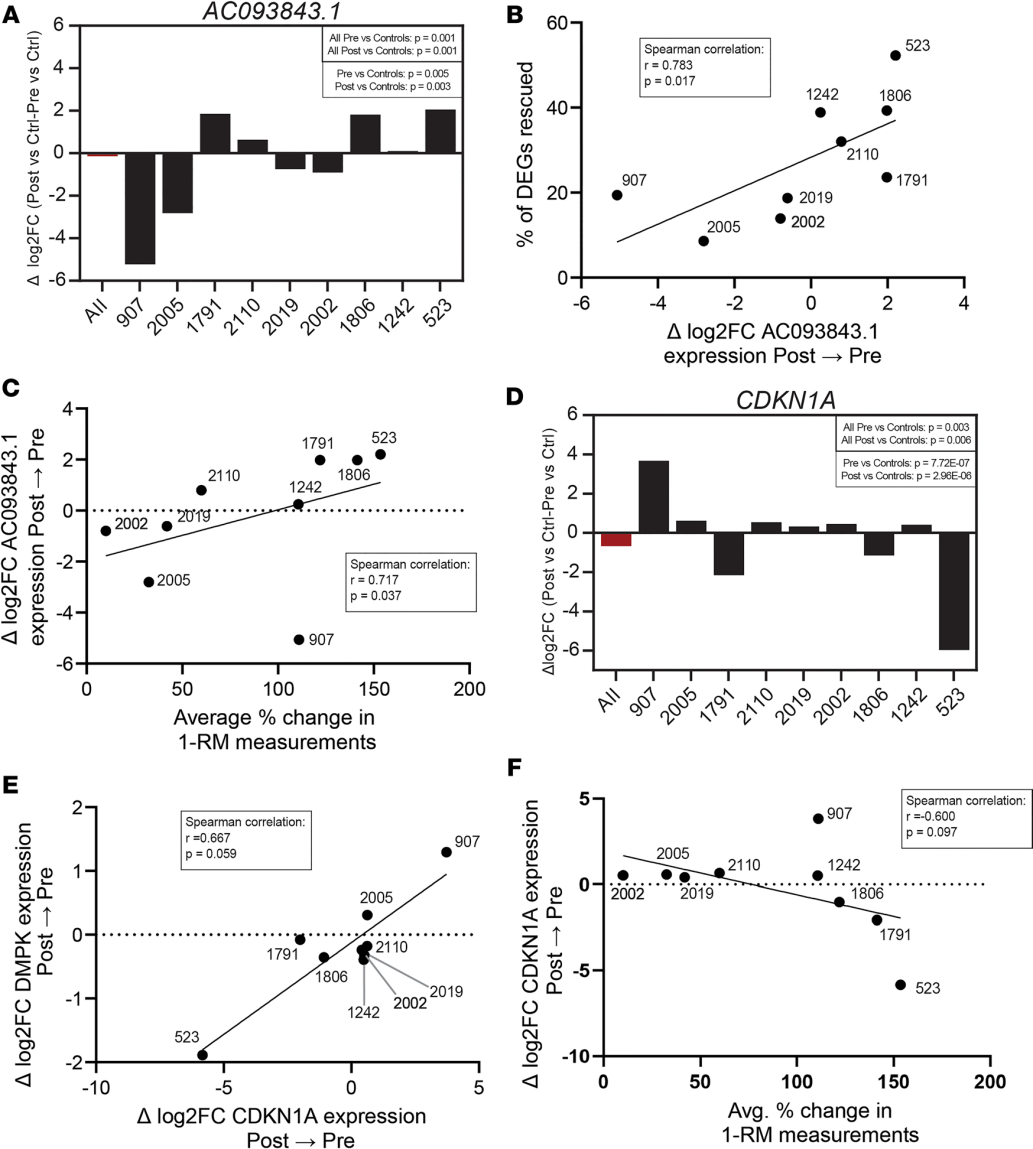
Expression of specific genes correlate with transcriptomic and clinical changes after strength training. (**A**) Change in expression of *AC093843.1* for each pre- to posttraining individual for individual (black bars) and grouped (red bar) versus unaffected controls. *P* values were calculated among pretraining individuals, posttraining individuals, grouped pretraining individuals, and grouped posttraining individuals using a quasi-likelihood F test. (**B**) Spearman correlation between the percentage of DEGs rescued and the change in expression of *AC093843.1* after strength training. (**C**) Spearman correlation between the change in expression of *AC093843.1* and the average percent change in 1-RM measurements after strength training. (**D**) Change in expression of *CDKN1A* before and after training for individuals as compared with unaffected controls. (**E**) Spearman correlation between the change in expression of *DMPK* and the change in expression of *CDKN1A* after strength training. (**F**) Spearman correlation between the change in expression of *CDKN1A* and the average percent change in 1-RM measurements after strength training. DEGs, differentially expressed genes; 1-RM, 1 repetition maximum.

Given the variability in response at the level of gene expression in both data sets, we analyzed our strength-training data to determine if any specific genes correlated to disease-specific transcriptomic measures (i.e., *DMPK* expression or percentage of DEGs rescued) or clinical improvements (i.e., 1-RM measurements) using a grouped descending analysis. For this analysis, we determined the overlapping rescued DEGs between the individual with the greatest clinical improvement (individual 523) and the individual with the next descending clinical improvement (individuals 1791, 1806, 907, and so on) until there were no longer overlapping rescued genes. Since there were no overlapping genes with 4 individuals, we examined the genes rescued among the top 3 individuals: *ENSG00000287690*, *AC093843.1*, *LRRC2-AS1*, *AC093225.1*, *ENSG00000287527*, and *FAM43B*. Among these 6 genes, only 1 gene, *AC093843.1*, showed significant correlations with both transcriptomic and clinical measures. *AC093843.1* showed a significant correlation with improvement at the level of gene expression (percentage of DEGs rescued) and clinical improvements (Figure 5, A–C) after strength training. This gene encodes a lncRNA with uncertain function, although its high expression levels have been associated with poor prognosis for ovarian cancer (32). We also examined whether this relationship was in the Mikhail et al. cycling training study (25) and found that changes in *AC093843.1* expression did not correlate with transcriptomic or clinical improvements after cycling training (Supplemental Figure 11, A–D). Only 1 other gene from the grouped descending analysis of our strength training data, *ENSG00000287527*, showed a significant correlation with the percentage of DEGs rescued (Supplemental Figure 12B) but not clinical outcome measures. This gene is another lncRNA with unknown function. lncRNAs can have a variety of functions, including gene expression regulation and cell development, although the association with either exercise or DM1 is unknown.

Given the limited number of overlapping genes in the grouped descending analysis, we reexamined the larger pool of genes rescued in the top 2 individuals with the greatest clinical improvement (74 genes) for overlap with DM-related genes. While many genes have previously been linked to DM, only 2 genes, *CDKN1A* and *DCLK1*, showed a statistically significant association within our data (Figure 5, D–F, and Supplemental Figure 13). *CDKN1A*, which encodes the p21 protein, has previously been shown to have increased expression in CUG-expansion cell lines (33) and in the gastrocnemius muscle of the HSA^LR^ DM1 mouse model (34). There is a moderate, albeit not statistically significant, correlation between *CDKN1A* expression with both transcriptomic and clinical measures (Figure 5, D–F). Strength training participants who experienced greater clinical benefit saw a decrease in *CDKN1A* expression (Figure 5D), suggesting a potential beneficial relationship between decreased *CDKN1A* expression and clinical outcomes for patients with DM1. We also found that there were substantial changes in *CDKN1A* expression after the cycling training program (Supplemental Figure 11E), and these changes inversely trend with transcriptomic improvements (percentage of DEGs rescued; Supplemental Figure 11F). However, changes in *CDKN1A* expression after cycling training did not correlate with *DMPK* expression or clinical outcomes (percent change in FEV_1_; Supplemental Figure 11, G and H), as they did after strength training. *DCLK1* is a microtubule-associated kinase involved in microtubule polymerization regulation (35), part of a muscle developmental gene network (36), and is misspliced in the brains of MBNL1-KO mice (35). There is a statistically significant correlation between *DCLK1* expression and percentage of rescued DEGs following strength training, and there is a trend, although not statistically significant, with changes in *DMPK* expression (Supplemental Figure 13). Understanding the correlation between the change in expression of these genes with transcriptomic and clinical changes may elucidate how changes in gene expression could be driving global transcriptomic improvements and how this may be connected to clinical improvements.

## Discussion

DM1 is the leading cause of adult-onset muscular dystrophy, which presents as a very heterogeneous disease, making effective diagnosis and treatment difficult. We have previously shown that a supervised 12-week strength-training program partially alleviates skeletal muscle impairments in male patients with DM1 (22). While exercise has been reported to ameliorate splicing dysregulation in mouse models (37–40), this same effect has yet to be reported in humans. Here we show that training can have a substantial, albeit heterogeneous, effect on the transcriptome of patients with DM1 and can partially rescue its hallmark splicing and gene expression defects. Global transcriptomic dysregulation was quantified by calculating both splicing and DGE dysregulation scores, and the dysregulation was used to measure global improvements after training for each individual. While changes at the level of splicing were consistent among all individuals except 1, changes at the level of gene expression were much more variable. Despite this variation, we observed that changes at the level of gene expression exhibited a strong correlation to clinical improvements (i.e., 1-RM measurement) after strength training. Analysis across our data set and reanalysis of a similar data set from a recent cycling training study (25) also demonstrated that individual analysis of patients with DM1 rather than grouped analyses may provide a more accurate overview of transcriptomic response. Our study reports that strength training, a nonpharmacological, low-cost, and accessible approach, has widespread effects that vary among individuals at the transcriptome level.

Our study suggests widespread effects at the transcriptome level, with both alternative splicing and gene expression, in response to strength training. This result contrasts with a recent Mikhail et al. cycling training study (25), which reported that aerobic exercise did not correct splicing defects in DM1. The aerobic training study also reported an association between a subset of small nucleolar RNAs (snoRNAs) that correlated with disease severity; however, our isolation methods did not purify these RNAs. Interestingly, we showed that participants in the aerobic training group had greater missplicing at the start of the study, and this could have contributed to differences in outcome measures between the 2 studies. While there are fundamental differences between the type of exercises, targeted systems and outcome measures between the 2 studies (discussed below), it is important to note that both studies reported clinical improvements specific to the type of stimulus (aerobic vs. strength training).

The reported lack of apparent transcriptomic change in the cycling training study was likely muted by group analysis, as reanalyzing using an individual analysis approach revealed that patients did exhibit improvements in splicing and gene expression dysregulation after cycling training. The individual level of response to aerobic training was greater in some individuals than others and, when grouped, resulted in an apparent lack of rescue at the level of splicing. We also observed minimal differences in both splicing and gene expression response after the strength-training program under grouped analysis. However, at the individual level, we observed distinct differences in their transcriptomic response before and after training. This variable response reinforces the heterogeneity of DM1 at a molecular level and in response to 2 types of training. There is considerable clinical heterogeneity among patients with DM1, including different clinical manifestations with different levels of severity. As a multisystemic disorder, symptoms vary greatly from one individual to the next, making it more efficacious to evaluate molecular level changes on an individual basis rather than as a group. Additionally, it is equally important to consider the importance of evaluating both clinical and transcriptomic effects within the same sex, as these sex-based differences are well documented at a clinical level (41) and, therefore, likely present at a molecular level as well. Only selecting male participants for this study allowed us to limit interindividual heterogeneity. Taken together, our analysis herein supports a benefit to evaluating strength training, or treatment, at an individual level.

The fact that we observed changes in expression at an individual level that are concordant with previously published expression changes in response to exercise suggests that the unique transcriptomic changes identified in our study are likely due to differences in molecular responses (e.g., changes in levels of splicing factors other than MBNL) to strength training as well as where these individuals are on the spectrum of DM1 severity. Individuals affected by DM1 can range from having a mild symptom (e.g., occasional myotonia) to being severely affected (e.g., wheelchair bound and severe muscle wasting), with missplicing being equally broad, making it challenging to determine the number of individuals needed to observe statistically significant changes at the level of splicing rescue in a cohort of patients with DM1.

The strength-training program that was the basis for this study (22) was designed to target the largest muscle group of the lower limb (i.e., the quadriceps) with specific exercises, in order to counteract muscle wasting and more largely improve day-to-day activity in patients with DM1. As such, the largest muscle (*vastus lateralis*) of the quadriceps muscle group was used for the biopsy analysis rather than the tibialis anterior, which is typically used in DM1 muscle studies. It is important to note that, even if DM1 is considered a progressive disease from distal to proximal, the strength of the quadriceps was significantly reduced at baseline in our cohort by reaching only 56.4% (ranging from 45.8% to 70.2%) of the predicted strength (22). Since this study is limited to *vastus lateralis* muscle biopsies, this same level of heterogeneity may not be present in other tissues. We observed considerable heterogeneity in splicing dysregulation for samples taken before and after the strength-training program as well as in the splicing events rescued by the strength-training program. This heterogeneity warrants further investigation to determine whether the *vastus lateralis* demonstrates an overall greater transcriptomic heterogeneity in patients with DM1 than other muscle groups. However, our data further highlight the fact that considerable individual heterogeneity of the DM1 transcriptome should be closely considered in patient studies, especially in the selection of splicing biomarkers for clinical trials. It was striking to observe in our data that more than 50% of the rescued skipped exon events were unique to each individual and that shared skipped exon events dropped dramatically across 4 or more individuals. In this context, it is not surprising that transcriptomic improvements were lost or muted when quantifying the molecular effects for all individuals on average. Likewise at the level of gene expression, the number of genes rescued, overrescued, or misrescued was highly varied from one individual to the next. This variability points to the benefit of quantifying improvement using a percent rescue metric as opposed to a stricter binary complete or not complete rescued metric. An approach using percent rescue will likely garner a more complete picture of transcriptomic changes and recognizes that partial improvements can still hold merit in understanding the molecular level effects of exercise.

The individual-based analysis of transcriptomic changes revealed interesting findings for specific patients with DM1. The 2 individuals with the smallest change and the largest change (decrease) in their DGE dysregulation score after strength training were individuals 907 and 523, respectively. These individuals also had the greatest increase and decrease in the expression of *DMPK*, respectively, suggesting that the change in expression of *DMPK* may play a role in the global dysregulation of gene expression in the participants with DM1. While this trend does not hold true for the Mikhail et al. aerobic training data (25), the changes in *DMPK* expression were not as pronounced after cycling training as after strength training. The mechanism behind these changes is difficult to unravel with our limited sample size but warrants further investigation in future studies. It is possible that altering *DMPK* expression may release transcription factors sequestered by toxic RNAs, thereby decreasing the global gene expression dysregulation. The sequestration of transcription factors by the CUG-expansion RNAs in DM1 has previously been reported to disrupt transcription (42). By decreasing the abundance of *DMPK* transcripts, fewer toxic RNAs are present to sequester transcription factors, which could free up factors to perform their normal functions in regulating transcription, thereby promoting normal gene expression patterns. The potential role that *DMPK* expression may play in regulating global gene expression patterns should be further investigated in the context of DM1, as it may have important implications in the disease and response to treatment.

Another variable of interest in respect to its effect on the transcriptome-level changes noted here is the age of the participants. The limited sample size of the original study (22) and lack of unaffected individuals of an age comparable with the participants with DM1 limits our ability to tightly control for age. Transcriptomic changes in skeletal muscle upon aging have previously been reported (43) and should be considered when evaluating transcriptomic changes that occur in patients with DM1 as compared with controls. To ensure our analysis was not skewed by differences in the average age between our groups (32 for control and 48 for DM1), we performed a Spearman correlation controlling for age for the correlation between the gene expression changes and the clinical improvement. We found that controlling for age differences only minorly strengthened the correlation coefficient (0.800 versus 0.837), suggesting that the overall trends noted from our analysis hold true despite the differences in age. These data reinforce that age should be considered to help control for confounding variables that may influence the transcriptome of both unaffected and individuals with DM1.

Our study identified 4 genes that potentially correlated with global transcriptomic changes and clinical improvements, including 2 lncRNAs (*AC093843.1* and *ENSG00000287527*) and 2 genes previously associated with DM1 (*CDKN1A* and *DCLK1*). The *CDKN1A* gene encodes the p21 cyclin dependent kinase inhibitor 1A protein, which has previously been shown to be increased in DM1 cell and animal models (33, 34). The p21 protein promotes cell cycle arrest in response to many stimuli, is part of a complex transcriptional network, and is implicated in multiple types of cancers (44). It is also a senescence factor that has been shown to be downregulated in response to exercise in animal models of chronic inflammatory myopathy (45). Given these diverse functions, it is difficult to ascribe a specific role to p21 in this study, but the connection does warrant further investigation. *DCLK1* is a microtubule-associated kinase involved in the regulation of microtubule polymerization (35), which has been shown to be part of a muscle developmental gene network (36) and is also misspliced in the brains of MBNL1-KO mice (35). It is possible that either connection to MBNL1 or muscle development is the source of association in our study, although further investigation is warranted to understand the specific roles. In contrast to the information on *CDKN1A* and *DCLK1*, very little is known about the 2 lncRNAs (*AC093843.1* and *ENSG00000287527*). Both are intergenic lncRNA, with *AC093843.1* (also known as *lnc-SLC4A3-9:1*) having been associated with poor prognosis in an ovarian cancer study (32) and no function or association ascribed to *ENSG00000287527* (also known as *lnc-PCDH1-1*). In general, lncRNAs regulate numerous molecular events, including gene expression, RNA processing, translational, and posttranslational processing through their interactions with DNA, RNA. and proteins (32). As with the other 2 genes, further investigation is required to determine if these lncRNAs have causative or correlation effects on exercise-induced transcriptomic changes.

### Strengths and limitations.

To the best of our knowledge, this study is the first to examine the effects of strength training in DM1 at the levels of alternative splicing and DGE. We attempted to account for DM1 heterogeneity using an individual analysis approach instead of a group analysis. While an individual analysis is often difficult to interpret and may reflect technical variation rather than true individual change, the individual-based approach in this study has the potential for identification of changes in data that would have been lost due to limited sample size and can also identify transcriptomic improvements masked in the recent Mikhail et al. data set (25). Even though we were not able to assess the effect of sex in this study as all our participants were male, we have an ongoing clinical trial (NCT05400629) addressing the effects of the same 12-week strength-training program in female participants with DM1 from the Saguenay–Lac-Saint-Jean region of Québec (Canada). When completed, analysis of the data sets between the 2 studies may clarify the effects of strength training in both male and female patients with DM1. Due to the limited muscle from biopsies, only 1 gene for DGE and 1 misspliced exon event were validated across 4 participants. Generally, these RT-PCR and qPCR results supported the RNA-Seq results, although some differences were observed. These differences may have arisen as RNA was reextracted from frozen sample as no RNA remained from the original RNA-Seq library preparations. Finally, large-cohort studies are especially difficult to conduct in rare diseases (e.g.,DM1) due to small sample population and lack of adequate funding for these studies. Therefore, the individual analysis approach described herein may help reveal molecular trends and biological mechanisms that will inform and aid future larger clinical trials.

### Conclusion.

We found that transcriptomic improvements were present in both alternative splicing and gene expression for male participants with DM1 after a 12-week strength-training program. While all participants had a clinical improvement, transcriptomic changes varied from one individual to the next. Most individuals had improvements at the level of splicing, but these improvements were largely unique to each individual. At the level of gene expression, individual improvements correlated strongly with clinical changes. Overall, this study suggests that exercise is a promising therapeutic for individuals with DM1; however, further investigation is needed to determine individual factors that drive an effective molecular-level therapeutic response to exercise.

## Methods

### Participants.

Participants with DM1 for the training program were previously recruited among patients followed at the Saguenay Neuromuscular Clinic of the Centre intégré universitaire de santé et de services sociaux du Saguenay–Lac-Saint-Jean (Quebec, Canada). Men with a genetic diagnosis of DM1, aged between 30 and 65 years, able to walk without assistance and to give their informed consent, and living in the Saguenay–Lac-Saint-Jean region were recruited; those with contraindications to maximal strength testing, training, or muscle biopsy excluded (22). Control subjects, for muscle biopsy only, were recruited among the team members, their families, and friends.

### Clinical assessments.

Functional evaluations used to assess the clinical effects of the training program were previously performed as described in Roussel et al. (22). Briefly, maximal isometric muscle strength was assessed using quantified muscle testing for the knee extensors and the 1-RM method for the following lower-limb strength exercises of the training program: leg extension, leg press, hip abduction, and squat. The 10-meter walk test (10mWT) and 30ssts repetitions were also documented. For all these clinical measurements, individual changes after the training program were reported in percentage compared with the preprogram value ([postprogram value – preprogram value]/preprogram value ***×*** 100) and analyzed using the standard error of measurement (SEMT) and the MDC. SEMT were calculated using the intraclass correlation (ICC; test-retest or intrarater) available in the literature for the DM1 population, with the SD obtained in our sample at baseline as follows: √(1 −*ICC*).

MDC were thereafter calculated using the formula MDC = 1.96 ***×*** SEMT √2 with individual changes deemed meaningful if they exceeded the MDC. See statistics section for SEMT values. General practitioners previously performed muscle biopsies of all DM1 training participants (before and after) and control (no training) subjects as previously described (22) with muscle samples frozen in liquid nitrogen at −80°C until further use.

### RNA-Seq library preparation.

RNA was extracting from frozen *vastus lateralis* samples of patients with DM1 (before and after training) and 6 unaffected controls (no training) using 1.5 mm zirconium beads (MidSci) and Zymo Quick-DNA/RNA microprep plus kit (Zymo Research) with on-column DNase treatment. RNA quality was checked via Fragment Analyzer (Advanced Analytical), and libraries were prepared via NEBNext Ultra II Directional RNA Library Prep Kit (Illumina) with NEBNext rRNA Depletion Kit (New England Biolabs) from at least 500 ng input RNA. Manufacturer protocols were used, with the following exceptions: 40***×*** adaptor dilutions were used, all bead incubations were done at room temperatures, a 4***×*** lower concentration of index primers was used; and 10 cycles of library amplification were performed. Libraries were pooled in equimolar amounts, quantified using the KAPA Library Quant Kit for Illumina (Kapa Biosystems), quality checked via fragment analyzer (Advanced Analytical), and sequenced (paired-end 75 bp) on the Illumina NextSeq 500 massively parallel sequencer (Albany Center for Functional Genomics).

### RNA-Seq analysis.

All samples were sequenced to a depth of at least 70 million reads and passed several quality control metrics via FastQC (version 0.11.9). The 2019 pretraining sample had a read depth twice that of other samples; therefore, to ensure the greater read depth would not affect downstream analyses, the FASTQ file for 2019 was downsampled using Seqtk (version 1.3) to a read depth similar to other samples. Samples 2182 and 1955 were excluded from our transcriptomic analysis due to poor RNA quantity and quality. Reads were mapped to the human reference genome (hg38) using STAR (version 2.7.9) (46). Changes in gene expression were quantified using edgeR (version 3.34.1) (47) in Rstudio v1.4.1106 (R version 4.1.2) (48). Genes with less than 10 counts in each samples were filtered out before downstream analysis. Significant DEGs were defined as those with log_2_FC > |2| and *P* < 0.05 for the strength-training program and log_2_FC > |1.5| and *P* < 0.005 for the cycling training program. Alternative splicing changes were quantified using rMATS (version 4.1.1) (49) to obtain PSI values for each misspliced event. Exons with less than or equal to 5 supporting reads were filtered out to remove transcripts with low read coverage. Significant skipped exon events were defined as those with ΔPSI > |0.2| and FDR < 0.05 (strength training) or ΔPSI > |0.05|, FDR < 0.05, and *P* < 0.0002 (cycling training).

### RT-PCR and qPCR analysis.

To validate the DGE analysis and misspliced exon events from the RNA-Seq studies, we performed new extractions of RNA from frozen *vastus lateralis* samples harvested from 4 patients with DM1 before and after the exercise program as described above. A total of 70 ng of RNA was reverse transcribed with the SuperScript IV Reverse Transcriptase (Thermo Fisher Scientific) to cDNA, which was used for qPCR analysis on an Applied Biosystems 7500 Real Time PCR system (Thermo Fisher Scientific) using Primers (IDT) for B2M (Hs.PT.58v.18759587), CDKN1A (Hs.PT.58.40874346.g), and PowerUp SYBR Green Master Mix (Thermo Fisher Scientific). RT-PCR reactions for CLASP1 exon 20 were done with Taq 2X Master Mix (New England Biolabs) using forward primer 5′-CAAAGTCTCCTCATCTTCGGGCACG-3′ and reverse primer 5′-GCTGGGACTGTGAAACCACTTTAGC-3′ and a 57°C annealing temperature. PCR products were visualized and quantified with the QIAxcel Advanced System (Qiagen). Each reaction was done in triplicate.

### Calculating percent rescue values.

Percent rescue values were calculated for all significant DEGs or misspliced skipped exon events when comparing pretraining samples with control samples, with control samples held at baseline. For gene expression, percent rescue was calculated as: (log_2_FC Pretraining – Posttraining/log_2_FC Pretraining – Average control) ***×*** 100. After the significant DEGs were identified and their corresponding log_2_FC values were obtained, the “Pretraining – Posttraining log_2_FC” values for the corresponding gene were obtained and percent rescue values were calculated. For alternative splicing, percent rescue was calculated as: (Pretraining PSI – Posttraining PSI/Pretraining PSI – Average control PSI) ***×*** 100. After the significant skipped exon events were identified and the “Pretraining – Average control ΔPSI” was obtained, the “Pretraining – Posttraining ΔPSI” values for the corresponding events were obtained and the percent rescue values were calculated. Rescued events for both gene expression and alternative splicing were defined as those with a percent rescue greater than 10% and less than 110%. Overrescue events were defined as those with a percent rescue greater than or equal to 110%. Misrescued events were defined as those with a percent rescue value less than –10%.

### Calculating dysregulation scores.

Gene expression dysregulation scores were calculated using the average absolute log_2_FC of all significant DEGs when comparing pretraining samples with control samples, with control samples held at baseline. To determine the posttraining gene expression dysregulation score, the same set of genes identified as significant in the pretraining versus controls comparison was used; however, the dysregulation score was calculated as the average absolute log_2_FC from the posttraining versus controls comparison. Similarly, the splicing dysregulation was calculated as the average absolute ΔPSI for all significant skipped exon events when comparing pretraining samples with control samples, with control samples held at baseline. The posttraining splicing dysregulation score was again calculated using the same set of skipped exon events identified as significant in the pretraining versus controls comparison; however, the splicing dysregulation score was calculated as the average absolute ΔPSI from the posttraining versus control comparison for these events.

### YGCY motif enrichment analysis.

To determine the enrichment of YGCY motifs (TGCT, TGCC, CGCT, and CGCC), the abundance of YGCY motif occurrences within the regulated exon, 250 bp upstream of the regulated exon and 250 bp downstream of the regulated exon for DM1 misregulated events, was compared with background events with similar nucleotide composition. For every 1 misregulated event, 5 background events were used for comparison. Enrichment values are reported as the log_2_(enrichment).

### Comparison with previously published exercise genes at an individual level.

We looked for genes reported to change in response to long-term, resistance exercise programs that also had a significant change in expression for all individuals in our 12-week strength-training program. The meta-analysis of Amar et al. was chosen, and it contained a comprehensive list of 114 genes reported to have changed expression levels due to long-term exercise intervention in skeletal muscle (26). Of those, 7 genes were highlighted within the meta-analysis, and their expression levels from 3 included studies that closely matched the criteria of ours were compared with expression levels for each individual in our study.

### Generating upset plots.

The upset plots showing the intersection in skipped exon events between individuals were generated using the ComplexHeatmap (version 2.10.0) R package.

### Correlations between transcriptomic and clinical improvements.

All correlations were calculated using the Spearman correlation coefficient. For performing Spearman correlations controlling for age, the ppcor (version 1.1) (50) R package was used to compute the Spearman *r* value and corresponding *P* value.

### Data availability.

Strength training data associated with this study have been deposited in Gene Expression Omnibus at GSE208639. Cycling training data published in Mikhail et al. are located at GSE184951. Data utilized from DMseq.org can also be accessed at GSE86356. Data from the Amar et al. (26) long-term exercise meta-analysis (labeled in the study as L_2, L_26, and L_28) used to compare previously published exercise genes with our training program are accessible at GSE59088 and GSE97084.

### Statistics.

For all clinical measurements, individual changes after the training program were reported in percentage compared with the preprogram value ([postprogram value – preprogram value]/preprogram value ***×*** 100) and analyzed using the SEMT. SEMT were calculated using the ICC (test-retest or intra-rater) available in the literature for the DM1 population, with the SD obtained in our sample at baseline as follows: SD baseline ***×*** (1-ICC). The following SEMT values were calculated: knee extensors strength = 3.77 Nm (51); comfortable walking speed = 0.0133 m/s; maximum walking speed = 0.0314 m/s (52); 30ssts = 0.41 (52). SEMT is not available for the 1-RM method. Changes in gene expression were quantified using edgeR, and *P* values were calculated by performing a quasi-likelihood F test to test for differential expression among any group as compared with unaffected controls held at baseline. Alternative splicing changes were quantified using rMATS, with *P* values calculated using a likelihood-ratio test. All correlations between transcriptomic and clinical improvements were calculated in GraphPad Prism version 9 using the Spearman correlation coefficient. For performing Spearman correlations controlling for age, the ppcor R package was used to compute the Spearman *r* value and the corresponding *P* value.

### Study approval.

The strength-training program study was previously approved by the Ethics Review Board of the Centre intégré universitaire de santé et de services sociaux of Saguenay–Lac-Saint-Jean (Québec, Canada), and all participants gave their written informed consent.

## Author contributions

EED performed RNA-Seq bioinformatics analysis, generated figures, wrote, and revised the manuscript. CL performed qPCR and RT-PCR experiments and wrote and revised the manuscript. LP performed RNA-Seq bioinformatics analysis and revised the manuscript. SS processed clinical samples, prepared RNA-Seq libraries, and performed RNA-Seq bioinformatics. CDL performed bioinformatics analysis. MPR supervised the training and collected and analyzed the clinical data. HKS conceived the experimental design and revised the manuscript. MH analyzed data and revised the manuscript. JDC designed the transcriptomic experimental approach, analyzed results, and revised the manuscript. ED conceived the clinical experimental design and analyzed the clinical data. JAB designed transcriptomic experimental approach, analyzed results, and revised the manuscript.

## Supplementary Material

Supplemental data

Supporting data values

## Figures and Tables

**Table 1:**
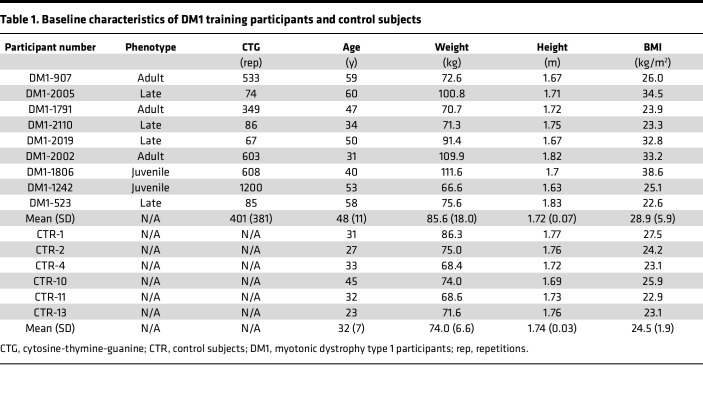
Baseline characteristics of DM1 training participants and control subjects

**Table 2:**
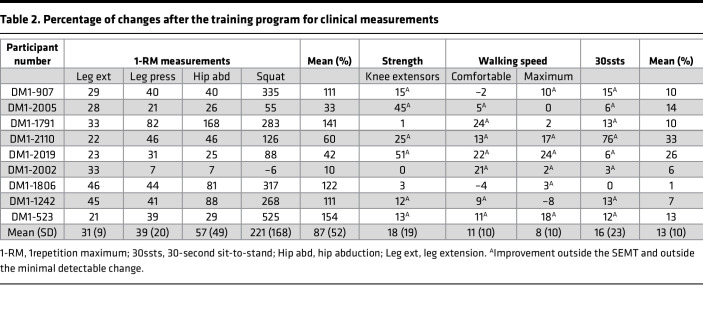
Percentage of changes after the training program for clinical measurements

## Version 1 06/15/2023

In-Press Preview

## Version 2 07/24/2023

Electronic publication
