# Supplementary material for: Individual transcriptomic response to strength training for patients with myotonic dystrophy type 1
Source: JCI Insight. 2023 Jul 24;8(14):e163856. doi: 10.1172/jci.insight.163856 (PMC10443797; doi:10.1172/jci.insight.163856)
Supplement: Supplemental data [file jciinsight-8-163856-s254.pdf]

(163856-INS-RG-RV-3) Davey et al. Supplemental Figures #1-13

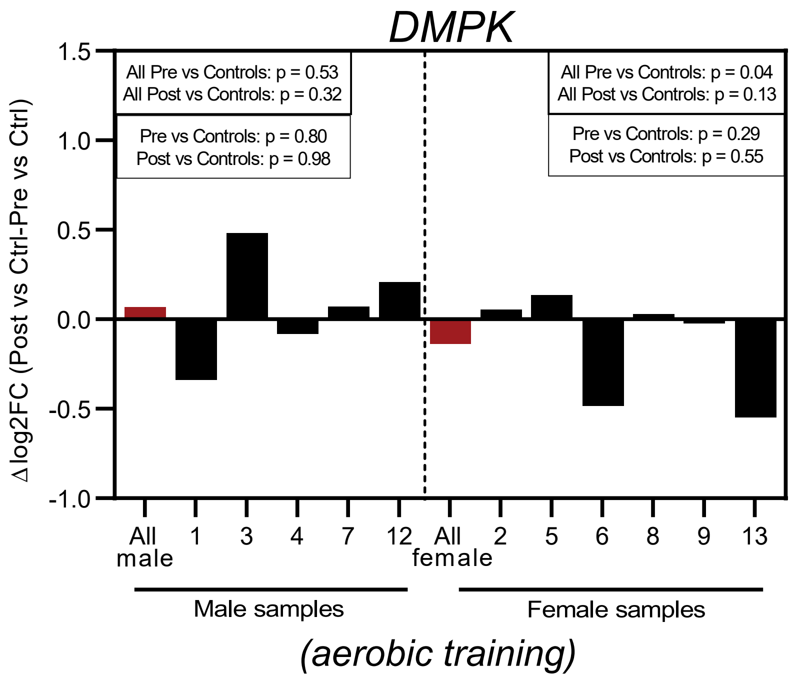

Supplemental Figure 1: Expression changes in DMPK after aerobic training. The change in expression of DMPK, measured as log2 fold change pre- to post-training for individuals (black bars) or grouped individuals (red bar) as compared to unaffected controls from Mikhail et al RNA-seq data (25) for each study participant, divided into male and female participants. P values were calculated among pre-training, post-training, grouped pre-training and grouped post-training individuals using a quasi-likelihood F-test.

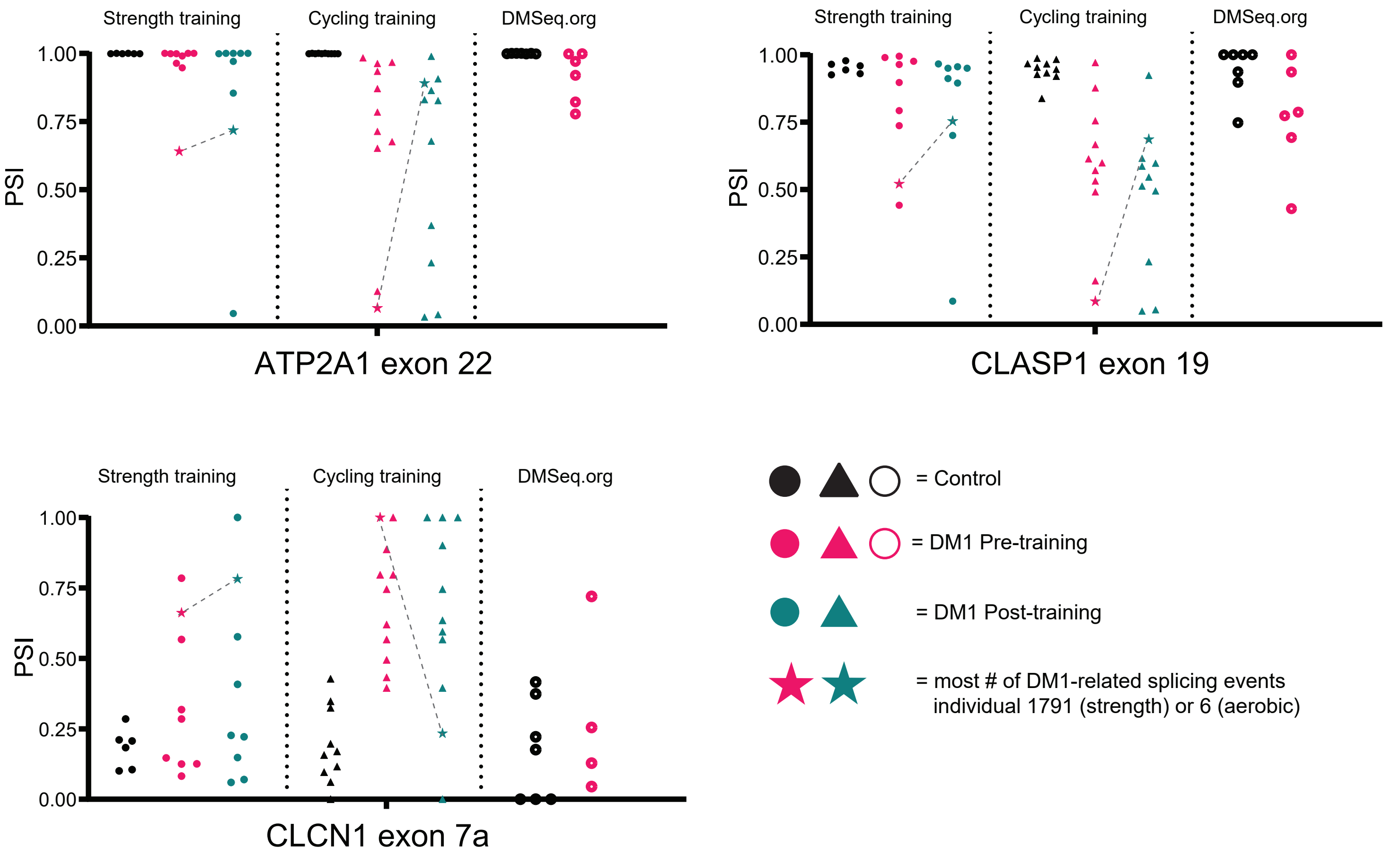

Supplemental Figure 2: Difference in DM1-specific spliced exon events for ATP2A1, CLASP1 and CLCN1 between RNA-seq datasets from strength training study, aerobic training study and DMseq.org (A) The percent spliced in (PSI) values of three DM1-associated splicing events for (A) ATP2A1 exon 22; (B) CLASP1 exon 20 (exon 19 in older genome annotations); and (C) CLCN1 exon 7a compared across control (black), pre-training (red); and post-training (green, when applicable) from datasets generated by strength training (left side, circles), Mikhail et al aerobic training (25) (middle, triangles), and DMseq.org (17) (right side, open circles). The individual that showed the greatest number of rescued events amongst the 46 DM1-related events (28) from strength training (left side, individual 1791) and aerobic training (middle, participant 6) are shown as stars. The grey dotted line marks the change in the PSI for those individuals from pre-training to post-training.

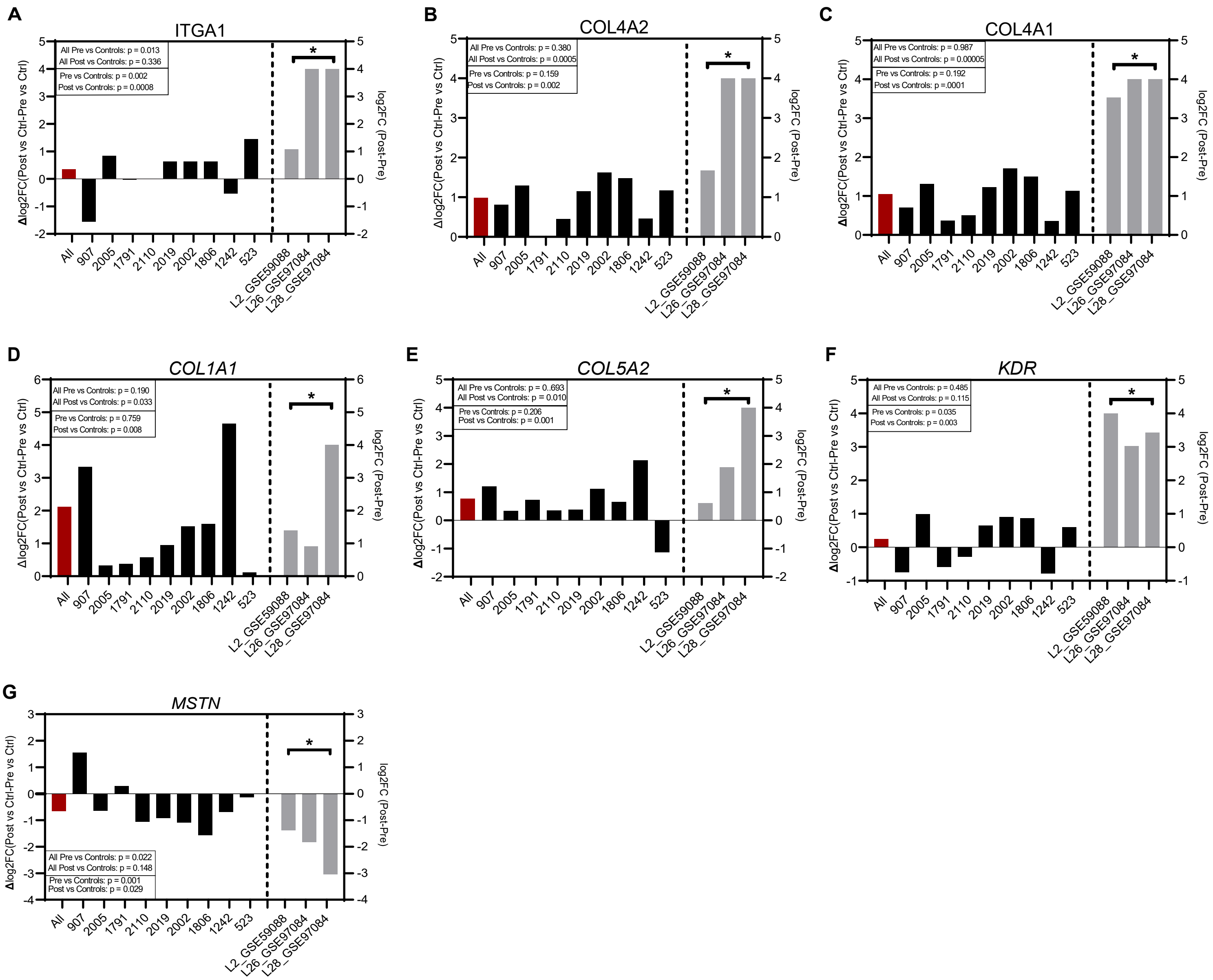

Supplemental Figure 3: Expression of genes previously reported to be significantly differentially expressed after long-term resistance exercise compared to strength training study (A-G) Changes in log2fold expression of seven genes after exercise compared between three 12-week resistance training cohorts from Amar et al (47). meta-analysis (gray bars), each individual from the 12-week strength training program (black bars), and all individuals from the 12-week strength training program grouped (red bar). P values for values in response to the strength training program were calculated using a quasi-likelihood F-test. Log2FC values for ITGA1 for 1791 and 2110 are 0.07 and 0.13, respectively. Log2FC value for COL4A2 for 1791 is 0.08.

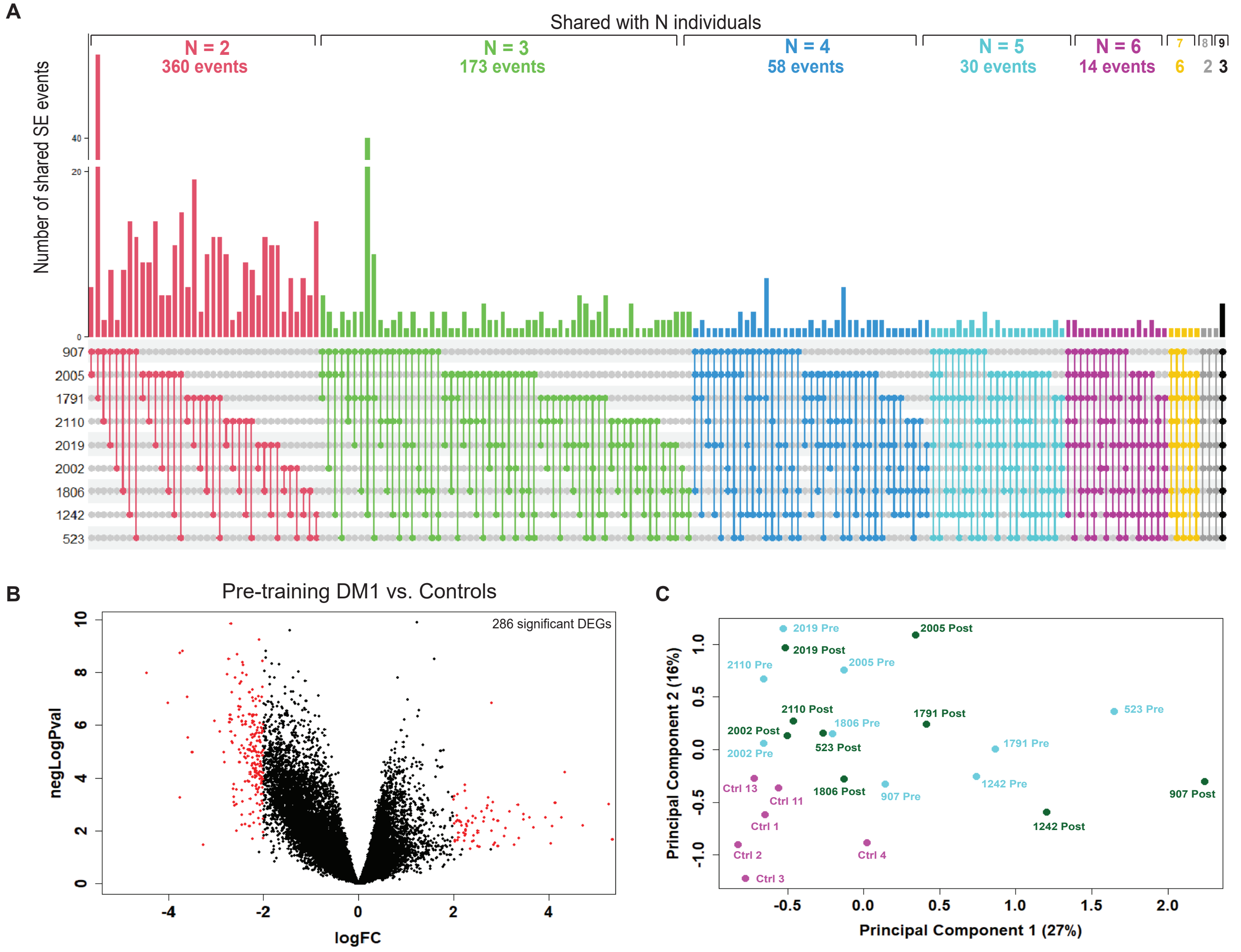

Supplemental Figure 4: DM1 participants prior to the strength training program recapitulate transcriptomic dysregulation known to be present in DM1. (A) The overlap of shared skipped exon (SE) events between strength training program DM1 participants relative to control participants, expressed as total shared events between N individuals, # of individual shared events within that total, and which individuals those events are shared between (connected dots). (B) Volcano plot showing significant up and downregulated genes in DM1 individuals, prior to the training program, as compared to controls (log2FC > |2| and $p < 0.05$). (C) PCA plot showing all pre-training, post-training and unaffected controls plotted against the two principal components accounting for the largest amount of variance between the samples Abbreviations: logFC: log2 of fold change, DEGs: differentially expressed genes.

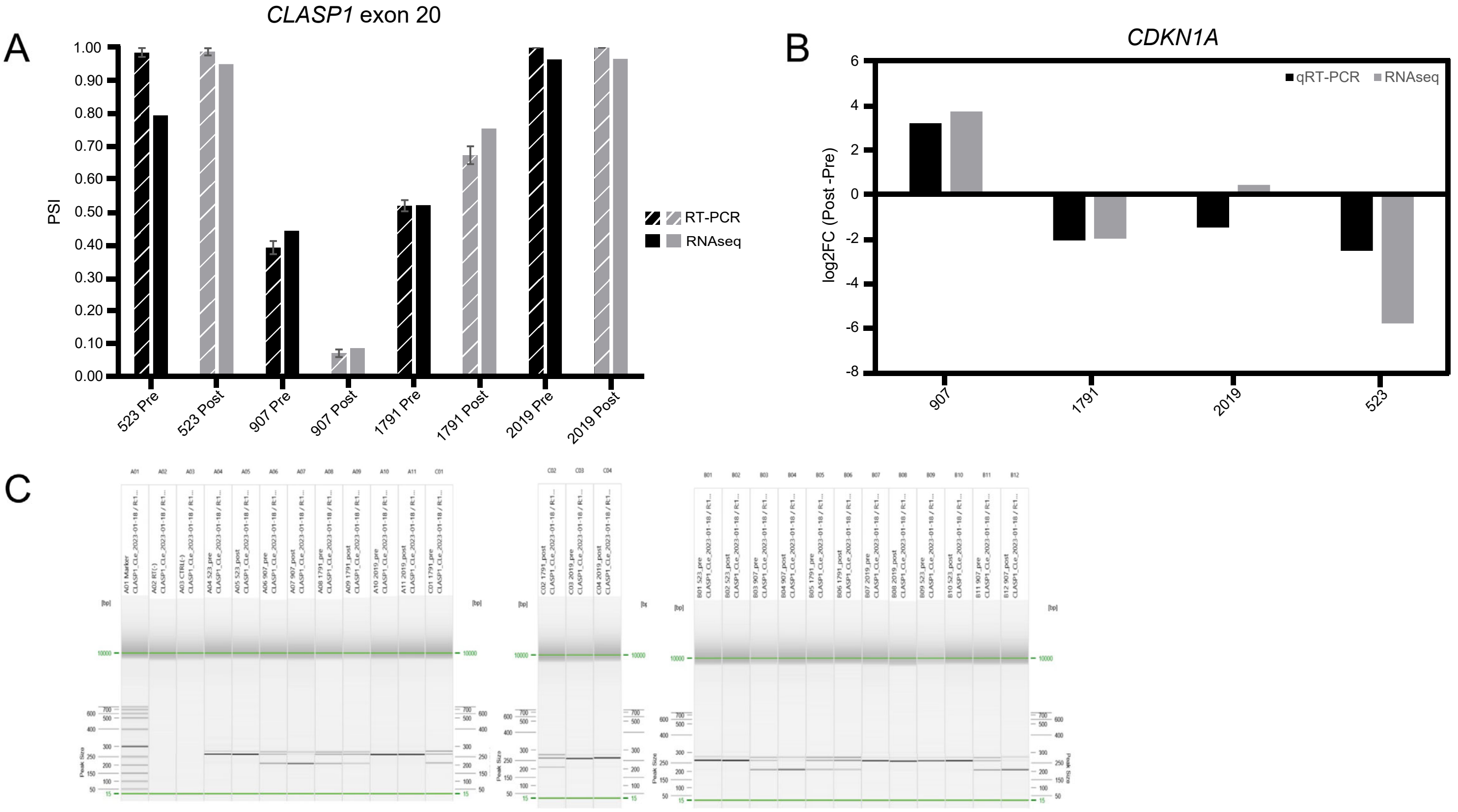

Supplemental Figure 5: Validation of RNAseq data. (A) RT-PCR validation of CLASP1 exon 20 (previously referred to as exon 19 in older genome annotations) exclusion before and after training. (B) qRT-PCR validation of CDKN1A DGE before and after training. (C) Gel images for RT-PCR validation of CLASP1 exon 20.

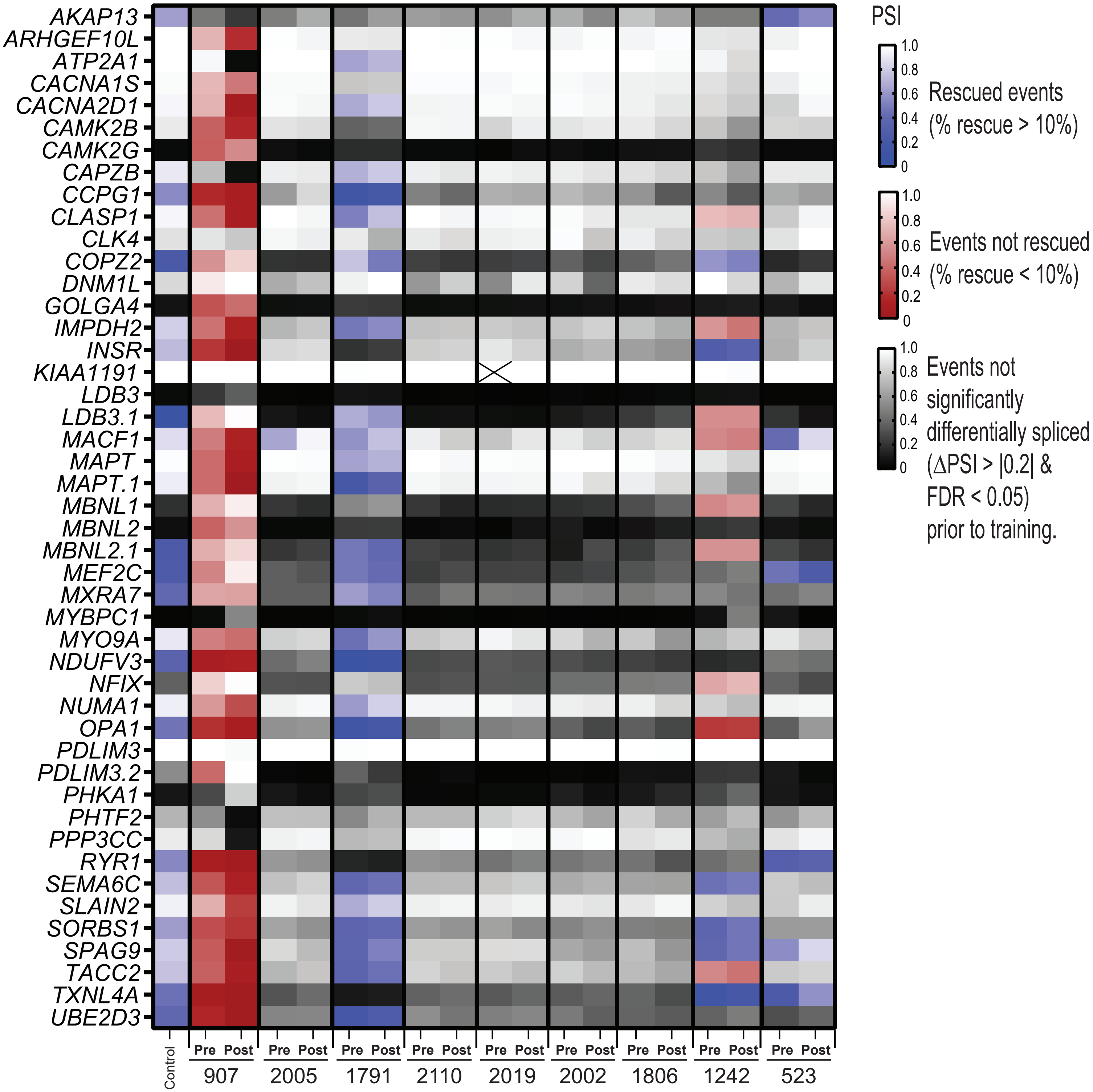

Supplemental Figure 6: Rescue of known DM1-specific skipped exon events in individuals after strength training. Heatmap of a panel of DM1-specific 46 skipped exon events that are good predictors of [MBNL]inferred levels (28) generated from strength training RNA-seq data. Statistically significant rescued (blue shading), not rescued (red shading) and not statistically significant (grey shading) events are illustrated. X boxes in table indicate no reads present for the splicing event. Abbreviations: FDR: false discovery rate, %: percentage, PSI: percent splicing in, pre: pre-training sample, post: post-training sample.

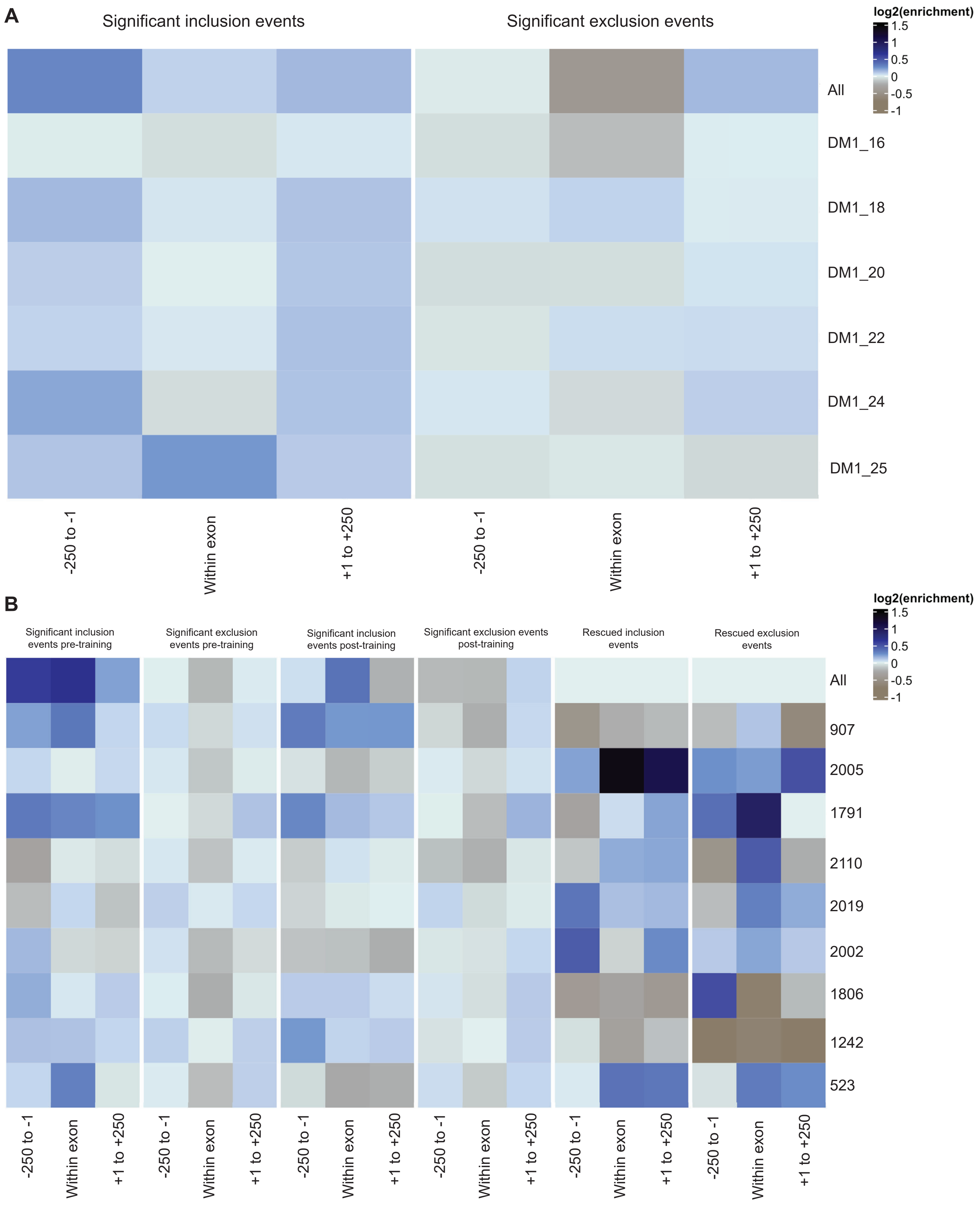

Supplemental Figure 7: Enrichment of YGCY motifs for DM1 mis-regulated skipped exon events. (A) Log2(enrichment) of YGCY motifs for significant inclusion events (ΔPSI > 0.2 and FDR < 0.05) and exclusion events (ΔPSI < -0.2 and FDR < 0.05) in male DM1 vastus lateralis samples from DMSeq.org, as compared to male unaffected controls. Enrichment was calculated 250 bp upstream of the mis-regulated exon, within the mis-regulated exon, and downstream of the mis-regulated exon.
(B) Log2(enrichment) of YGCY motifs for significant inclusion events (ΔPSI > 0.2 and FDR < 0.05) and exclusion events (ΔPSI < -0.2 and FDR < 0.05) in male DM1 vastus lateralis samples pre-training and post-training. Enrichment was also calculated for inclusion and exclusion events rescued by the training program.

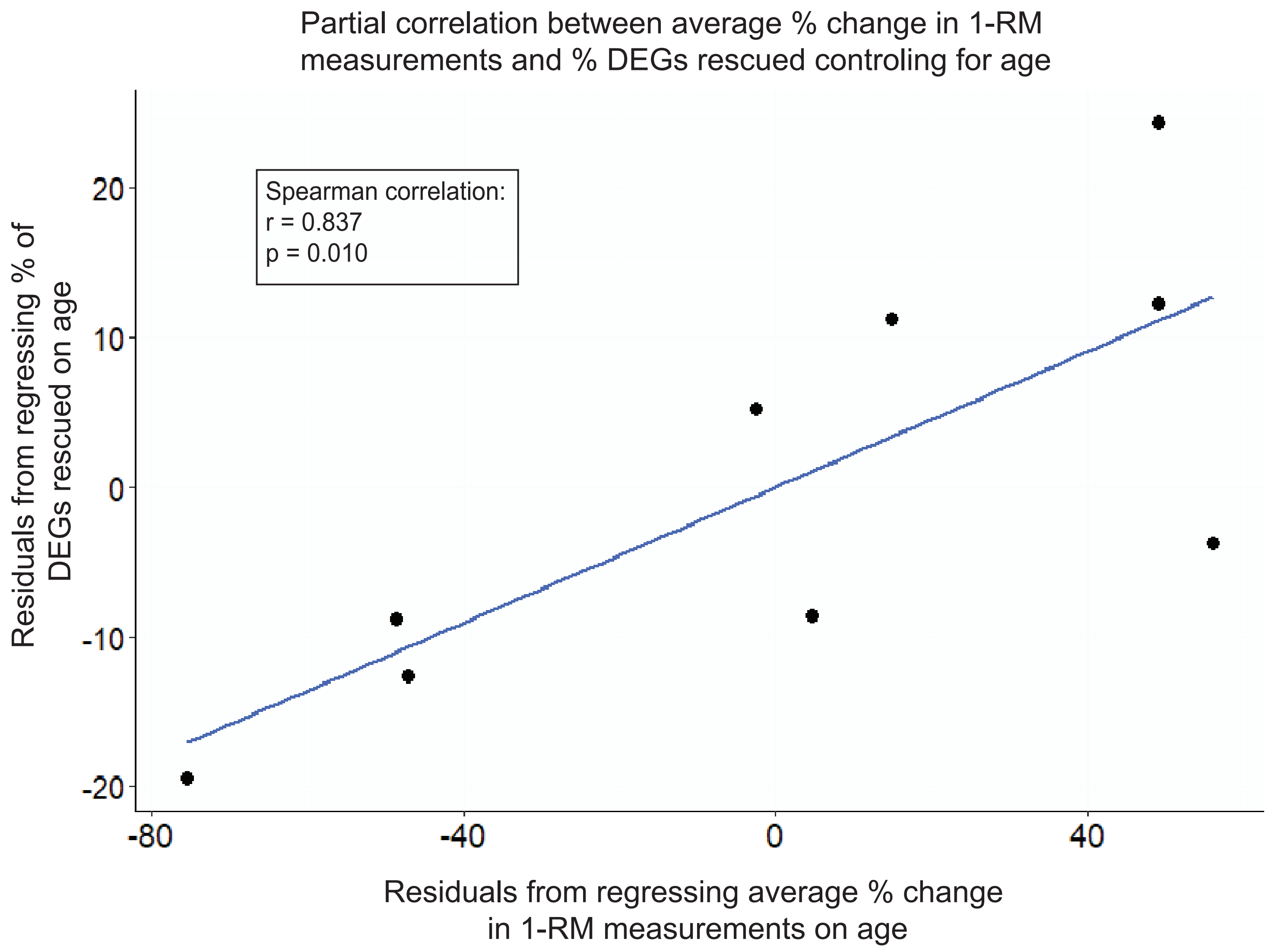

Supplemental Figure 8: Age-adjusted spearman correlation between clinical measurements and differentially expressed genes after strength training. Spearman correlation between the average percent change in 1-RM measurements (clinical outcome measures) and the percentage of DEGs rescued after strength training. Abbreviations: 1-RM: one-repetition maximum, DEGs: differentially expressed genes.

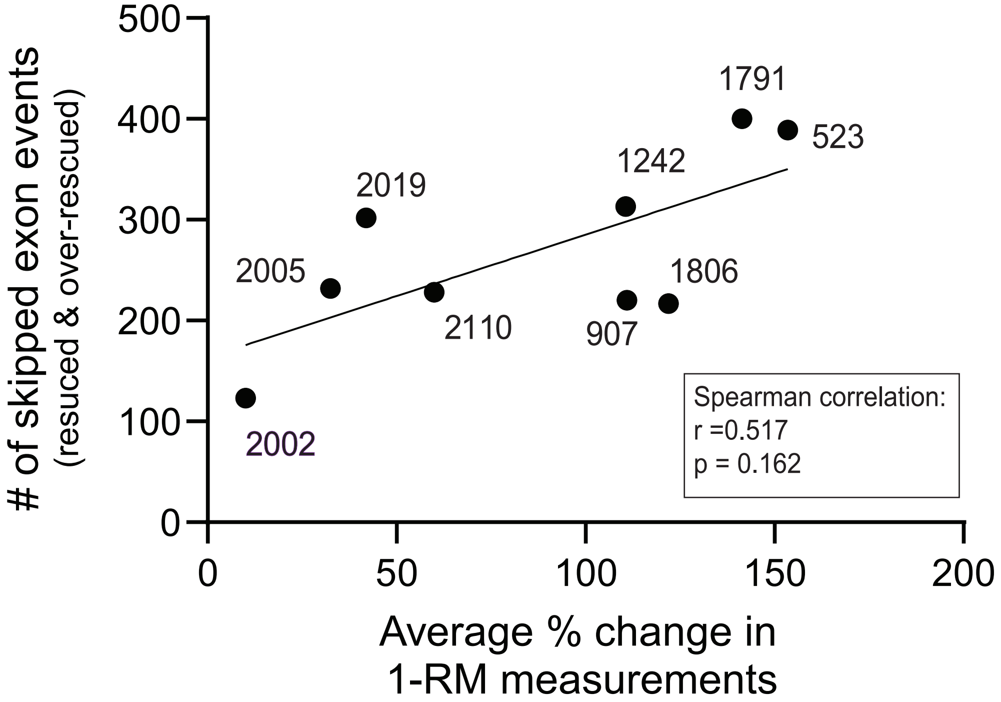

Supplemental Figure 9: Correlation between splicing rescue and clinical measurements after strength training. Spearman correlation between the number of rescued and over-rescued events and average percent change in 1-RM measurements (clinical improvement). Rescued are defined as those events where the percent spliced in (PSI) value shifts more than 10% but less than 110% while over-rescued are defined as events with PSI greater than or equal to 110% beyond control levels. Abbreviations: %: percentage, 1-RM: one-repetition maximum, SE: skipped exon.

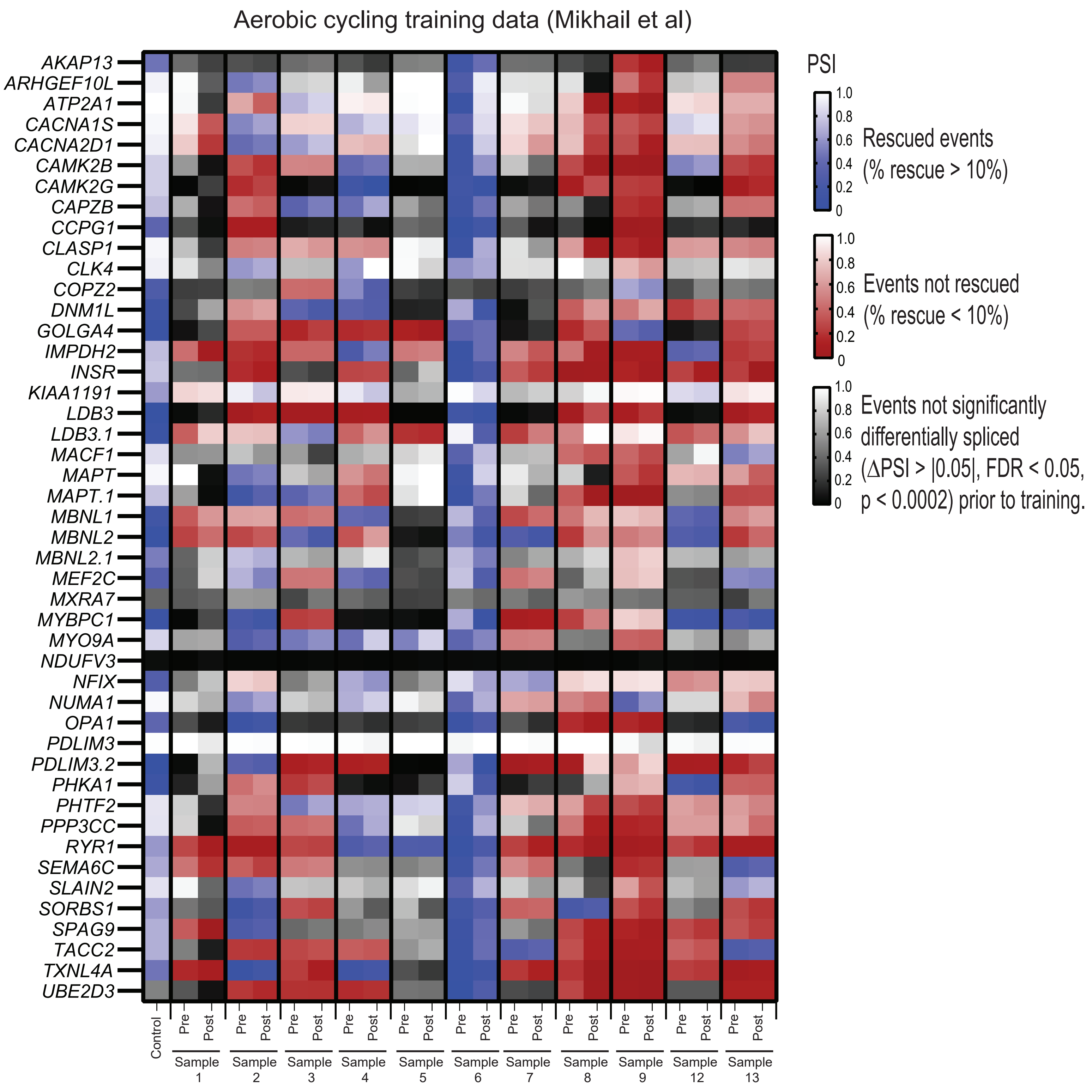

Supplemental Figure 10: Rescue of known DM1-specific skipped exon events in individuals after Mikhail et al aerobic training. Heatmap of a panel of DM1-specific 46 skipped exon events that are good predictors of [MBNL]inferred levels (28) generated for each individual in the Mikhail et al aerobic training RNA-seq dataset (25). Statistically significant rescued (blue shading), not rescued (red shading) and not statistically significant (grey shading) events are illustrated. Abbreviations: FDR: false discovery rate, %: percentage, PSI: percent splicing in, pre: pre-training sample, post: post-training sample.

Aerobic cycling training data (Mikhail et al)

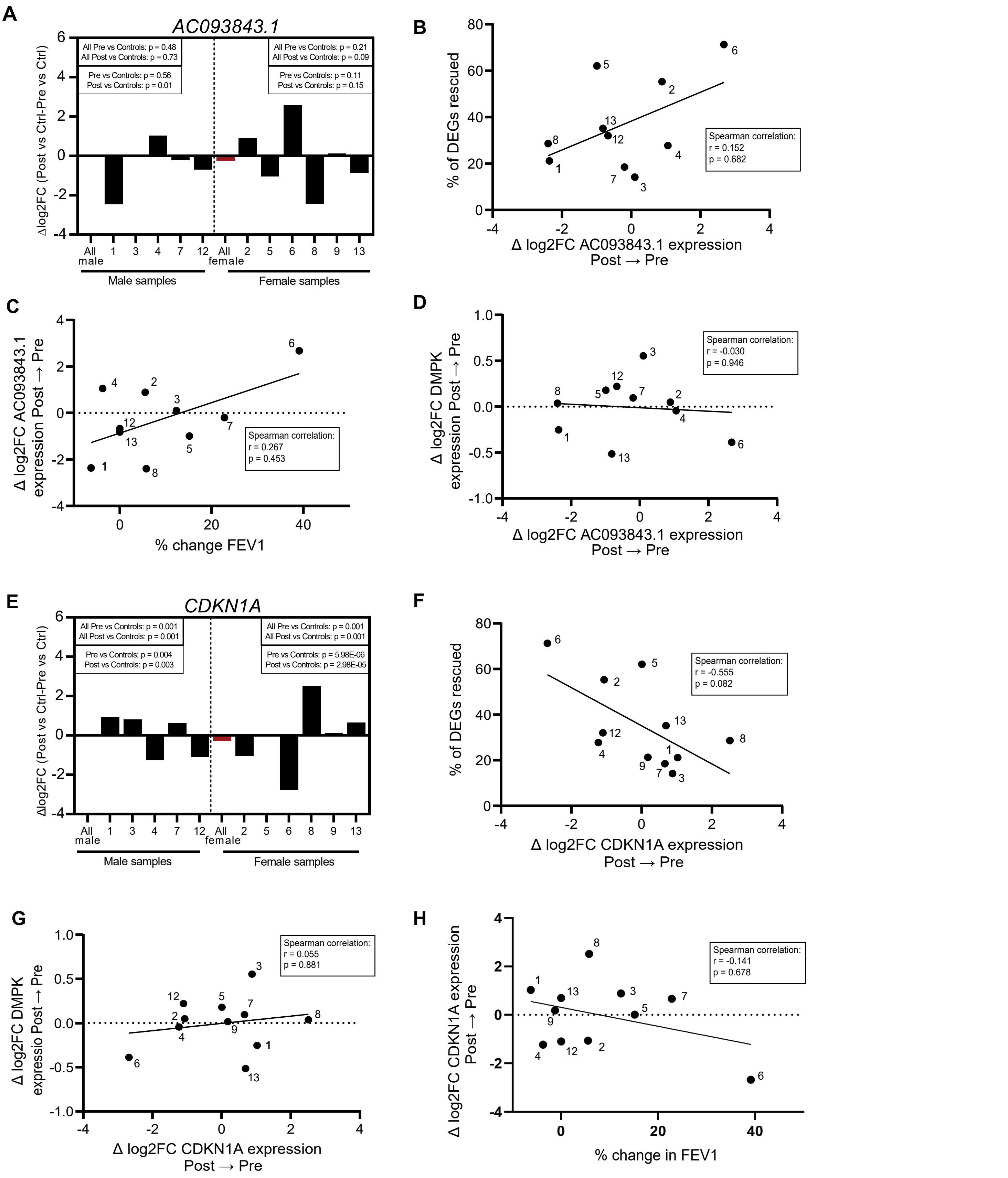

Supplemental Figure 11: Expression of lncRNA AC093843.1 and CDKN1A correlated with transcriptomic and clinical changes after Mikhail et al aerobic training. (A) Changes in expression of AC093843.1 for each individual pre- to post-training (black bars) and all individuals grouped (red bar) compared to unaffected controls, divided by sex. P values were calculated using a quasi-likelihood F-test among pre-training, post-training, grouped pre-training and grouped post-training individuals for each sex. (B) Spearman correlation between the number of rescued DEGs and the log2Fold change in expression of AC093843.1 following aerobic training. Rescued is defined as those genes where the expression value shifts more than 10% but less than 110% in the direction of control samples. (C) Spearman correlation between the change in log2fold expression of AC093843.1 and the percent change in FEV1 after aerobic training. (D) Spearman correlation between the change in log2fold expression of DMPK and the change in log2fold expression AC093843.1 after aerobic training. (E) Changes in expression of CDKN1A pre- to post-aerobic training for individuals (black bars) and all individuals grouped (red bar) compared to unaffected controls, divided by sex. P values were calculated using a quasi-likelihood F-test. (F) Spearman correlation between the number of rescued DEGs and the log2Fold change in expression of CDKN1A following aerobic training. (G) Spearman correlation between the change in log2fold expression of DMPK and the change in log2fold expression CDKN1A after aerobic training. (H) Spearman correlation between the change in log2fold expression of CDKN1A after aerobic training and the percent change in FEV1.

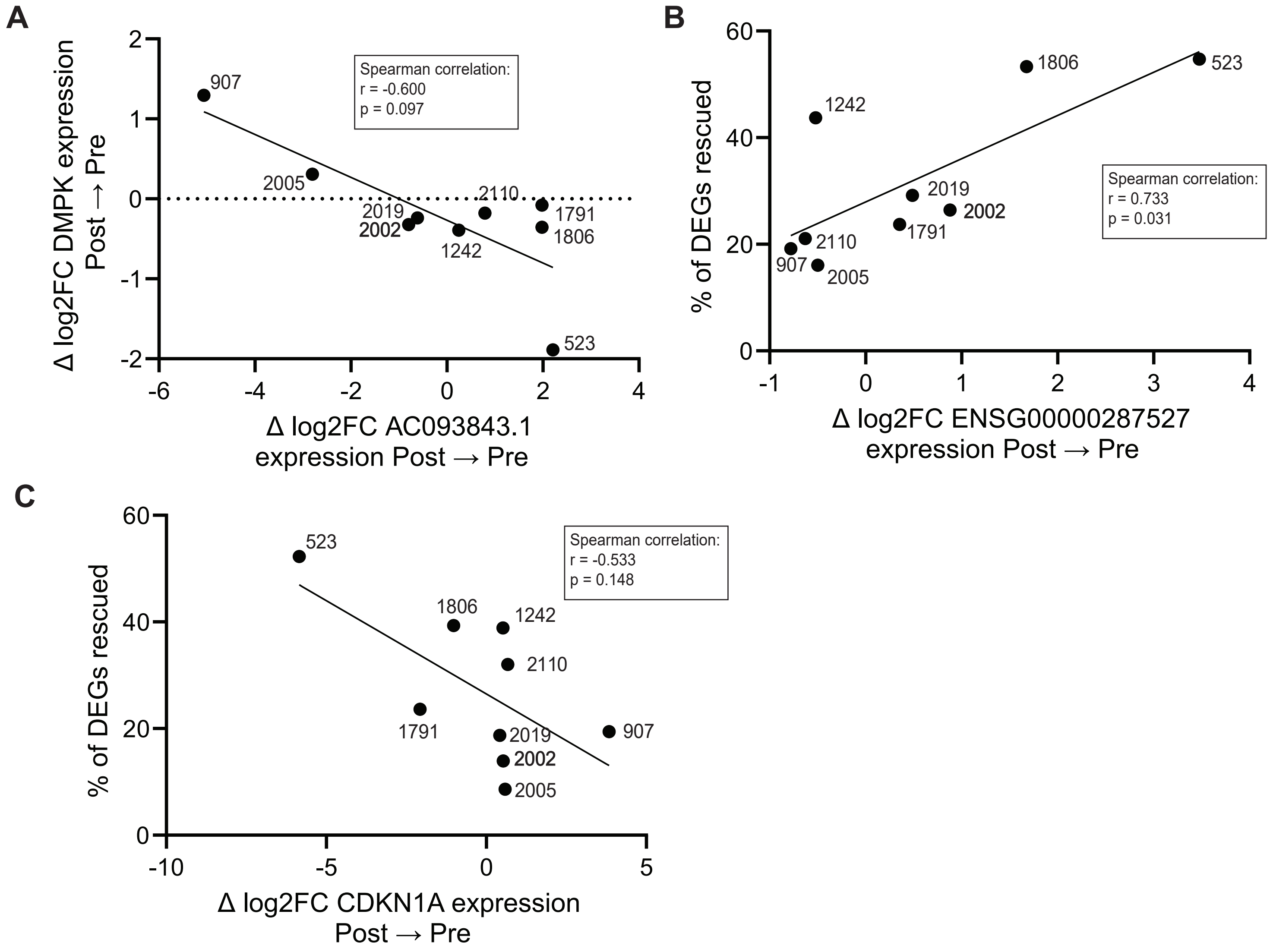

Supplemental Figure 12: Expression of AC093843.1, ENSG00000287527, and CDKN1A after strength training program (A) Spearman correlation between the change in log2fold expression of DMPK and the change in log2fold expression of AC093843.1 after strength training. (B&C) Spearman correlation between the number of rescued DEGs and the log2Fold change in expression of (B) ENSG00000287527 or (C) CDKN1A, following strength training. Rescued is defined as those genes where the expression value shifts more than 10% but less than 110% in the direction of control samples. Abbreviations: %: percentage, DEGs: differentially expressed genes

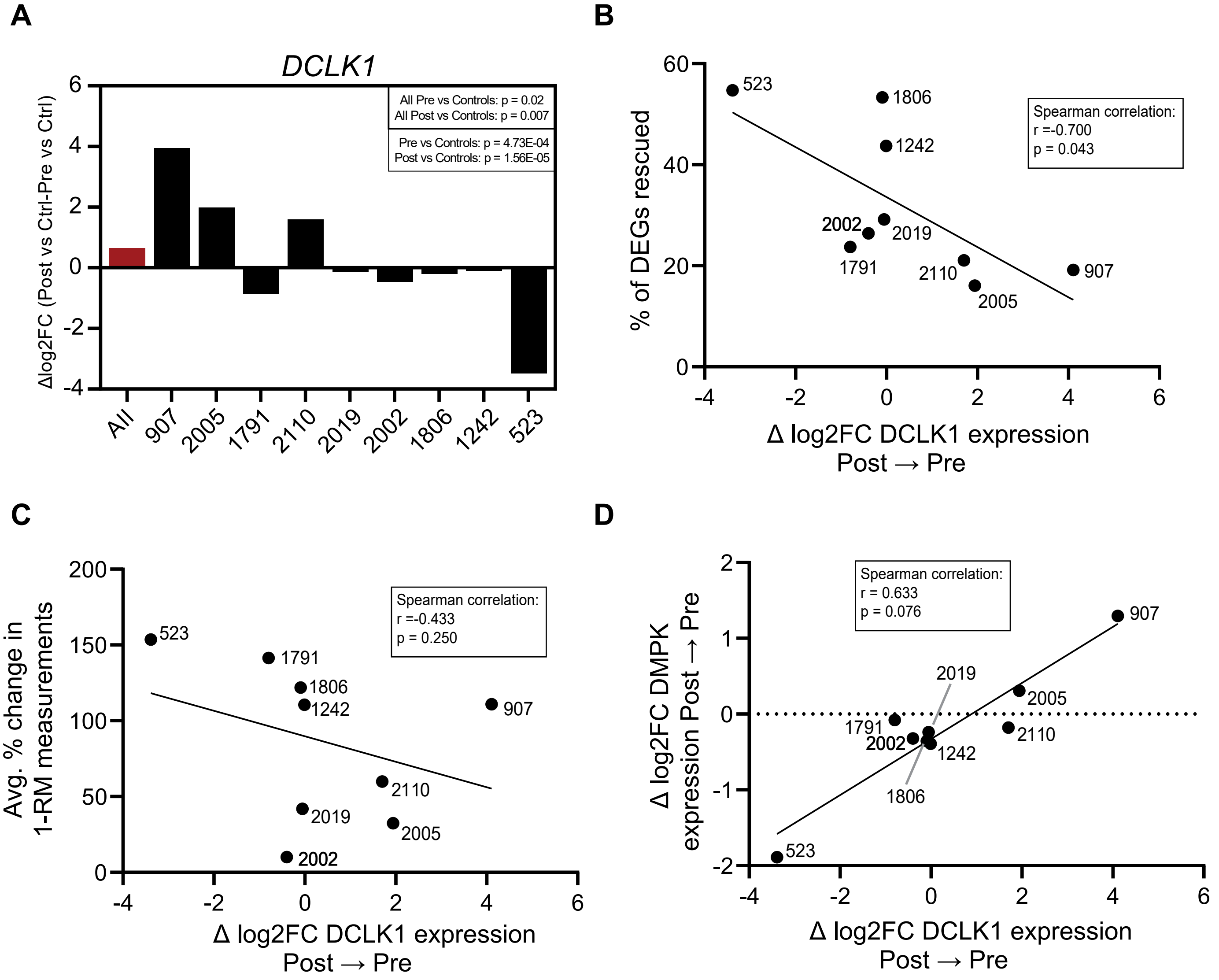

Supplemental Figure 13: Expression of DCLK1 gene with transcriptomic and clinical changes after strength training. (A) Changes in expression of DCLK1 pre- to post-strength training for individuals (black bars) and all individuals grouped (red bar) compared to unaffected controls. P values were calculated using a quasi-likelihood F-test. (B) Spearman correlation between the number of rescued DEGs and the log2Fold change in expression of DCLK1 following strength training. Rescued is defined as those genes where the expression value shifts more than 10% but less than 110% in the direction of control samples. (C) Spearman correlation between average percent change in 1-RM measurements and the change in log2fold expression of DCLK1 after strength training. (D) Spearman correlation between the change in log2fold expression of DMPK and the change in log2fold expression CDKN1A after strength training. Abbreviations: %; percentage, DEGs: differentially expressed genes.
